# Prevalence of the Antibiotic Resistance of *Salmonella typhi* and *Salmonella paratyphi* in Pakistan: A Systematic Review and Meta-analysis

**DOI:** 10.1093/ofid/ofaf131

**Published:** 2025-03-11

**Authors:** Aftab Ullah, Muhammad Shabil, Saud A Abdulsamad, Asif Jan, Abdulghani A Naeem, Haseen Ullah, Mudassir Khattak, Zakiullah

**Affiliations:** Department of Pharmacy, University of Peshawar, Peshawar, Pakistan; Department of Pharmacy, Abasyn University Peshawar, Peshawar, Pakistan; Center for Global Health Research, Saveetha Medical College and Hospital, Saveetha Institute of Medical and Technical Sciences, Saveetha University, Chennai, India; Medical Laboratories Techniques Department, AL-Mustaqbal University, Hillah, Iraq; Basic Sciences Department, College of Science and Health Professions, King Saud bin Abdulaziz University for Health Sciences, Jeddah, Saudi Arabia; King Abdullah International Medical Research Center, Jeddah, Saudi Arabia; Department of Pharmacy, University of Peshawar, Peshawar, Pakistan; District Headquarter Hospital, Charsadda, Pakistan; Basic Sciences Department, College of Science and Health Professions, King Saud bin Abdulaziz University for Health Sciences, Jeddah, Saudi Arabia; King Abdullah International Medical Research Center, Jeddah, Saudi Arabia; Department of Pharmacy, University of Peshawar, Peshawar, Pakistan; Department of Pharmacy, Abasyn University Peshawar, Peshawar, Pakistan; Department of Pharmacy, University of Peshawar, Peshawar, Pakistan

**Keywords:** antibiotic resistance, antibiotic susceptibility, enteric fever, *Salmonella paratyphi*, *Salmonella typhi*

## Abstract

**Background:**

Antibiotic resistance to *Salmonella* is a significant threat to public health globally, particularly in low- and middle-income countries such as Pakistan. This study reviews the existing literature to determine the pooled prevalence of antibiotic resistance among *Salmonella typhi* and *Salmonella paratyphi* strains across Pakistan in the past decade, including the emergence of extended-spectrum β-lactamase.

**Methods:**

Six databases were searched for studies published from January 2014 to December 2024. Studies were screened for relevance, and data were extracted on antibiotic susceptibility among human *S typhi* and *S paratyphi* isolates. Their quality was assessed per the Joanna Briggs Institute checklist. A random effects model was employed by R statistical software (version 4.4) to calculate the pooled resistance rates.

**Results:**

Thirty-one studies met the inclusion criteria after full-text screening. The analysis revealed significant resistance rates to commonly used antibiotics for *S typhi*, including nalidixic acid (92%; 95% CI, 88%–95%), ampicillin (80%; 95% CI, 66%–89%), ciprofloxacin (64%; 95% CI, 48%–77%), azithromycin (7%; 95% CI, 3%–16%), and meropenem (2%; 95% CI, 1%–3%), with notable variations across cities, and for *S paratyphi*, such as nalidixic acid (91%; 95% CI, 82%–96%), ampicillin (34%; 95% CI, 21%–50%), ciprofloxacin (51%; 95% CI, 25%–77%), azithromycin (4%; 95% CI, 1%–12%), and meropenem (2%; 95% CI, 1%–5%). In *S typhi*, 29% and 25% of patients had multidrug resistance (95% CI, 21%–41%) and extensive drug resistance (95% CI, 12%–44%), respectively; corresponding rates for *S paratyphi* were 9% (95% CI, 2%–28%) and 2% (95% CI, 1%–7%).

**Conclusions:**

The findings revealed the alarming prevalence of antibiotic-resistant *Salmonella* in Pakistan and the need for updated treatment guidelines. Public health strategies must focus on improving antibiotic use and developing alternative treatment options to mitigate the rising threat of resistant *Salmonella* strains. Continued research, policy intervention, and national and international cooperation are essential to safeguard public health and ensure effective management of enteric fever.

## Teaser

This systematic review and meta-analysis reveals high antibiotic resistance in Salmonella typhi and Salmonella paratyphi in Pakistan, with alarming resistance to first-line antibiotics. The emergence of multidrug-resistant (MDR) and extensively drug-resistant (XDR) strains highlights the urgent need for updated treatment guidelines, improved antibiotic stewardship, and enhanced vaccination efforts in endemic regions.

Antimicrobial resistance (AMR) has been recognized as a global health crisis, with projections suggesting that by 2050, an AMR-related infection will result in the death of 1 person every 3 secondsl [1, 2]. However, these figures might slightly overestimate the mortality rate, with recent studies such as the *Global Burden of Bacterial Antimicrobial Resistance 1990–2021* [3] providing more conservative estimates based on updated data. The World Health Organization (WHO) lists *Salmonella* as a priority pathogen for antibiotic resistance, underscoring its significant global threat [4]. Particularly, Pakistan has been at the forefront of the emergence of extended-spectrum β-lactamase (ESBL) and extensive drug-resistant (XDR) *Salmonella typhi*, making this study highly relevant. *S typhi* typically enters the body by ingesting contaminated food and water and causes enteric fever. *S typhi* and *Salmonella paratyphi A* and *B* (rarely *S paratyphi C*) cause enteric fever, a serious bacterial infection primarily transmitted through contaminated food and water [5]. Symptoms of enteric fever include fever with chills, nausea, diffuse abdominal pain, rash, anorexia, splenomegaly, hepatomegaly, constipation or diarrhea, and relative bradycardia on physical examination [6].

Global distribution of enteric fever is estimated to be 11 to 20 million cases annually, with South Asia having the highest ratio [7]. Enteric fever continues to be a major public health problem and is endemic in Pakistan. Specifically, typhoid fever poses a significant risk in low- and middle-income countries and is caused by *Salmonella enterica* serovar *typhi* (*S typhi*) [8]. In addition to being the fourth-most prevalent cause of death worldwide, typhoid fever causes 128 000 to 161 000 deaths annually [7], with Pakistan having the third-highest typhoid rate. Among children aged 2 to 5 years in Pakistan, the incidence rate is 573.2 per 100 000 per year [4].

Antibiotics are the primary treatment for typhoid fever; however, *S typhi* and *S paratyphi* are now exhibiting resistance to these antimicrobials. Multidrug resistance (MDR) is an emerging health challenge for a growing nation worldwide [9]. Between 1970 and 1980, multiresistant *S typhi* (MDR *S typhi*) emerged, and these bacterial strains were sensitive to quinolones but resistant to first-line treatments such as ampicillin, chloramphenicol, and cotrimoxazole [10]. The incidence of MDR *S typhi* increased from 34.2% to 48.5% between 2001 and 2006, and the percentage of patients resistant to ciprofloxacin increased from 1.6% to 64.1%. Consequently, ceftriaxone, a third-generation cephalosporin, was prescribed. Yet, the first case of XDR *S typhi* was documented in 2016 in Sindh province, Pakistan. Azithromycin and carbapenems are the only available treatments for XDR typhoid [7]. Azithromycin has replaced the third-generation cephalosporins because of its cost-effectiveness [6]. The resistance of azithromycin has reportedly increased in Bangladesh: 32 of 2519 isolates of *S typhi* identified between October 2016 and July 2018 were found to be resistant to the antibiotic. Drug-resistant *S typhi* infections result in higher rates of morbidity and mortality, as well as an economic burden from longer hospital stays and higher costs of therapies [11]. Typhoid vaccinations have been added to the WHO list of essential drugs for priority diseases due to increased drug-resistant strains in endemic areas [7].

The increasing incidence of antibiotic-resistant typhoid in Pakistan has been attributed to various factors, including the limited availability of sanitary facilities and clean water, as well as poor public knowledge of the disease [12]. In addition, the lack of vaccination and restriction on antibiotics have been important causes. It is a common practice to prescribe antibiotics empirically (ie, before a definitive diagnosis), particularly for potentially severe infections such as enteric fever, especially in regions with high endemicity [13].

Because no systematic review or meta-analysis was available on this topic, this evidence-based study was conducted to fill that gap. Therefore, this study aims to determine the prevalence of antibiotic resistance, including MDR and XDR status, against *Salmonella* species in the 4 provinces of Pakistan over the last decade.

## METHODS

The study was conducted and reported per the PRISMA guidelines (Preferred Reporting Items for Systematic Reviews and Meta-analyses; Supplementary Table 1). Articles were deduplicated and screened with rayyan.ai (web-based software).

### Search Strategy

Embase, Google Scholar, PubMed, Science Direct, Web of Sciences, and the local database PakMediNet were comprehensively searched to identify and find potentially relevant studies published from January 2014 to December 2024. The search was conducted on 15 July 2024. The search terms used were as follows: (“*Salmonella typhi*” OR “typhoid”) AND (“*Salmonella paratyphi* OR paratyphoid”; “antibiotic resistance” OR “antimicrobial resistance” OR “drug resistance”) AND (“Pakistan”). The filtered years ranged from 2014–2024 (Supplementary Table 2).

### Inclusion and Exclusion Criteria

All original articles (cross-sectional, prospective, or retrospective studies) that identified the prevalence of antibiotic resistance in *Salmonella* species isolates in Pakistani human populations were considered for the study. Research with unclear findings, case reports, conference presentations, reviews, and research written in languages other than English were excluded. Given that *S typhi* is a human-restricted pathogen, the inclusion criteria focused exclusively on studies involving human populations, and data related to nonhuman populations were not considered (Supplementary Table 3).

### Screening

The citations were collected from different data sources and entered into the citation classification program (Rayyan) with duplicate articles removed at identification. After exclusion of any irrelevant article, 2 reviewers (A. U. and M. S.) separately scrutinized the titles and abstracts. In the second step, they independently evaluated the entire texts of the articles. A third reviewer was consulted to resolve any discrepancies between the reviewers regarding the inclusion of studies.

### Data Extraction

Two researchers (A. U. and M. K.) independently extracted data from potentially relevant studies: first author, publication year, study design, sample size, patient demographics (age, sex), type of diagnostic test used for antibiotic susceptibility, prevalence of resistance to specific antibiotics, geographic location within Pakistan, and years of data collection. All potential discrepancies among reviewers were resolved through discussion to reach a consensus.

### Quality Assessment

The quality of the studies was evaluated per the Joanna Briggs Institute checklist (Supplementary Table 4). Each article in the review was independently rated and analyzed by 2 reviewers (A. U. and M. S.), with differences being resolved through discussion.

### Statistical Analysis

Statistical software R (version 4.4.0) was used to conduct the meta-analysis and generate the forest plots for the present study. The random effects model was used with an inverse variance method to pool the number of samples with resistance and the total samples tested for each drug separately. *S typhi* and *S paratyphi* were analyzed separately. Subgroup analysis was performed by province or city. The *I*^2^ index was used to evaluate the potential for heterogeneity among studies. To assess the publication bias, funnel plots were developed.

### Antibiotic Susceptibility Testing

Antibiotic susceptibility testing was performed via the Kirby-Bauer disc diffusion method, also referred to as KBD, following the standard protocols. Both terms refer to the same testing method used to determine the antibiotic resistance profile of the isolates.

In this study, MDR was defined as resistance to at least 3 classes of antibiotics commonly used for treating enteric fever: ampicillin, ciprofloxacin, and chloramphenicol.

XDR was defined as resistance to at least 1 agent in all but ≤2 antimicrobial categories routinely used to treat enteric fever, such as third-generation cephalosporins, macrolides, and carbapenems.

## RESULTS

### Literature Search

**Figure 1.**
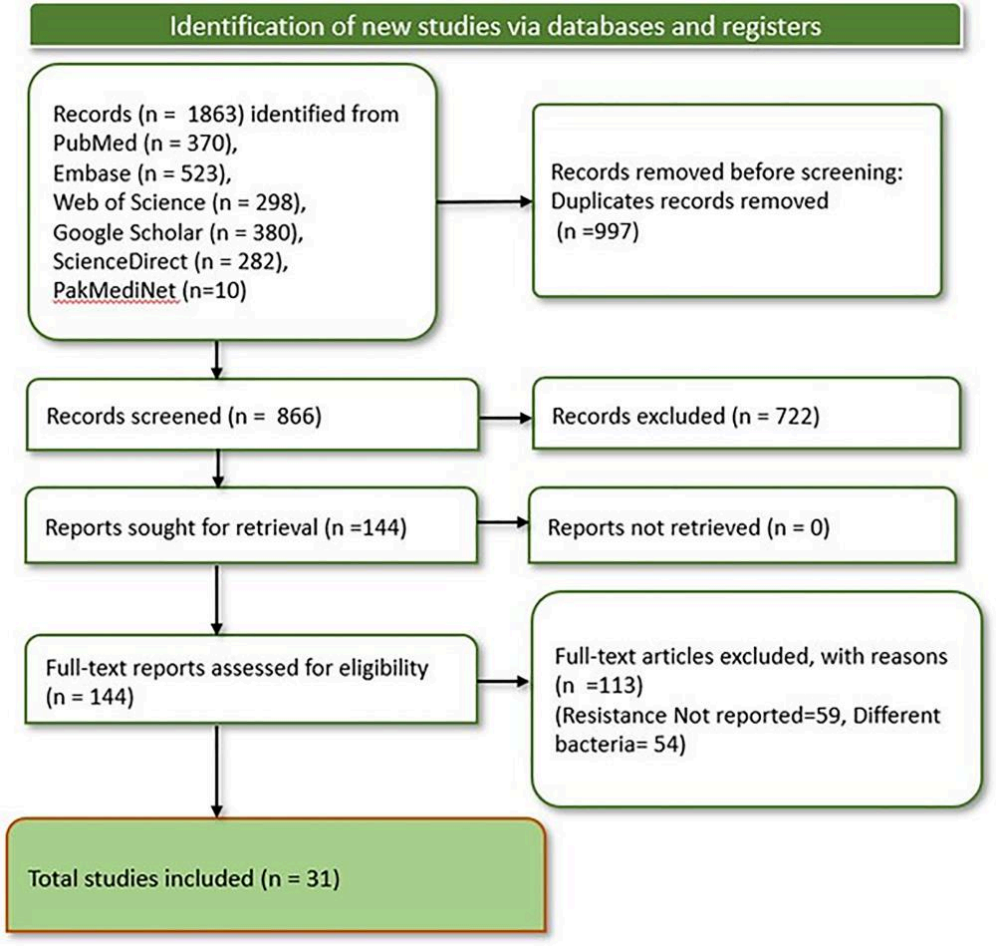
PRISMA flowchart depicting the process of the final set of the included studies.

A total of 1863 records were obtained by screening 6 databases, 997 of which were identified as duplicates. After removal of these duplicates, primary screening of the remaining 866 studies was performed by title and abstract. Full-text screening of 144 studies was also carried out. A total of 113 studies were excluded: 59 did not mention the prevalence of resistance, and 54 had different species of bacteria. Hence, 31 articles (Table 1) [9, 14–43] were deemed eligible for inclusion in this meta-analysis and systematic review (Figure 1).

**Table 1.** Characteristics of the Included Studies

Study	Design	City	Duration	No.	Mean Age, y	Male	Antibiotic Susceptibility Test
Yousaf 2024 [14]	Observational	Peshawar	Sep 2021–Aug 2022	504	12.92	182	KBD
Umair 2020 [17]	Cross-sectional	Islamabad	2012–2018	664	25.5	440	Blood culture
Aslam 2021 [18]	Cross-sectional	Lahore	Jan 2019–Mar 2020	50	22.5	34	KBD
Saeed 2019 [16]	Retrospective	Islamabad	Jan 2015–Dec 2018	598	12.8	24	KBD
Saeed 2020 [15]	Cross-sectional	Lahore	2018	82	18.5	52	PCR
Hussain 2022 [19]	Cross-sectional	Kharia (District Gujrat, Punjab)	Jan 2019–Sep 2020	76	22.37	41	KBD
Aslam 2024 [20]	Retrospective	Lahore	1 Jan 2017–31 Dec 2022	90	36.79	42	…
Khan 2024 [21]	Prospective	Peshawar	1 Nov 2020–31 Dec 2020	74	15.5	56	KBD
Laghari 2019 [22]	Cross-sectional	Jamshoro (Sindh)	Jul–Dec 2018	275	5.3	163	KBD
Ullah 2024 [23]	Cross-sectional	Peshawar	2017–2023	3137	25.8	2044	KBD
Ejaz 2022 [24]	Cross-sectional	Lahore	1 Jan–31 Dec 2021	62	12.9	44	KBD
Anjum 2021 [25]	Cross-sectional	Karachi	1 Sep 2018–28 Feb 2019	76	5.7	39	KBD
Zahid 2022 [9]	Cross-sectional	Lahore	1 Apr 2021–30 Sep 2021	50	28.1	36	Blood culture
Batool 2021 [27]	Retrospective	Lahore	Sep 2018–Feb 2019	182	16.8	99	Blood culture
Ahmad 2022 [28]	Retrospective	Swat	Jun 2020–Dec 2020	120	11.5	84	KBD
Sattar 2020 [29]	Observational	Sialkot	1 Jan–30 Jun 2019	55	23.1	34	KBD
Hussain 2019 [30]	Prospective	Karachi	1 Jan–31 Dec 2018	291	24.8	237	KBD
Izhar 2020 [31]	Cross-sectional	Rawalpindi	Jan–Dec 2019	179	15.4	99	KBD
Ahmad 2020 [32]	Retrospective	Peshawar	Jun 2019–May 2020	168	4.76	94	KBD
Sherazi 2023 [33]	Cross-sectional	Peshawar	Oct 2019–Oct 2021	71	6.6	107	Blood culture
Khan 2023 [34]	Cross-sectional	Lahore	Jan–Dec 2019	100	15.6	58	KDB
Baig 2023 [35]	Cross-sectional	Peshawar	Jul 2020–Jan 2021	105	8.48	70	Gram staining and API20E
Shah 2024 [36]	Prospective	Lahore	2 y	530	15.68	320	Blood culture
Ejaz 2022 [24]	Retrospective	Lahore	1 Jan–31 Dec 2021	62	13.2	302	KBD
Zahid 2022 [9]	Cross-sectional	Lahore	1 Apr 2021–30 Sep 2021	50	28.5	32	Microbiological testing of typhoid slides
Latif 2019 [37]	Retrospective	Lahore	Jan 2018–Apr 2019	98	11.8	57	Mueller-Hinton agar, KBD
Rasheed 2023 [38]	Descriptive study	Lahore	1 Jan 2021–31 Dec 2022	235	25.5	195	Blood culture
Shabbir 2023 [39]	Cross-sectional	Sukkur	Aug 2020–Jan 2021	55	15.6	40	Gram staining, culture, and biochemical testing
Shaikh 2019 [40]	Retrospective	Lahore	Jan–Dec 2017	465	5.4	223	Blood culture
Zahid 2022 [41]	Observational	Lahore	2019–2020	180	9	100	Biochemical, serologic, and PCR-based molecular characterization
Ahmad 2023 [42]	Observational	Peshawar	Nov 2020–15 May 2021	62	6	28	Blood culture

Abbreviations: KBD, Kirby-Bauer disk diffusion; PCR, polymerase chain reaction.

### Prevalence of Resistance to *S typhi*

**Figure 2.**
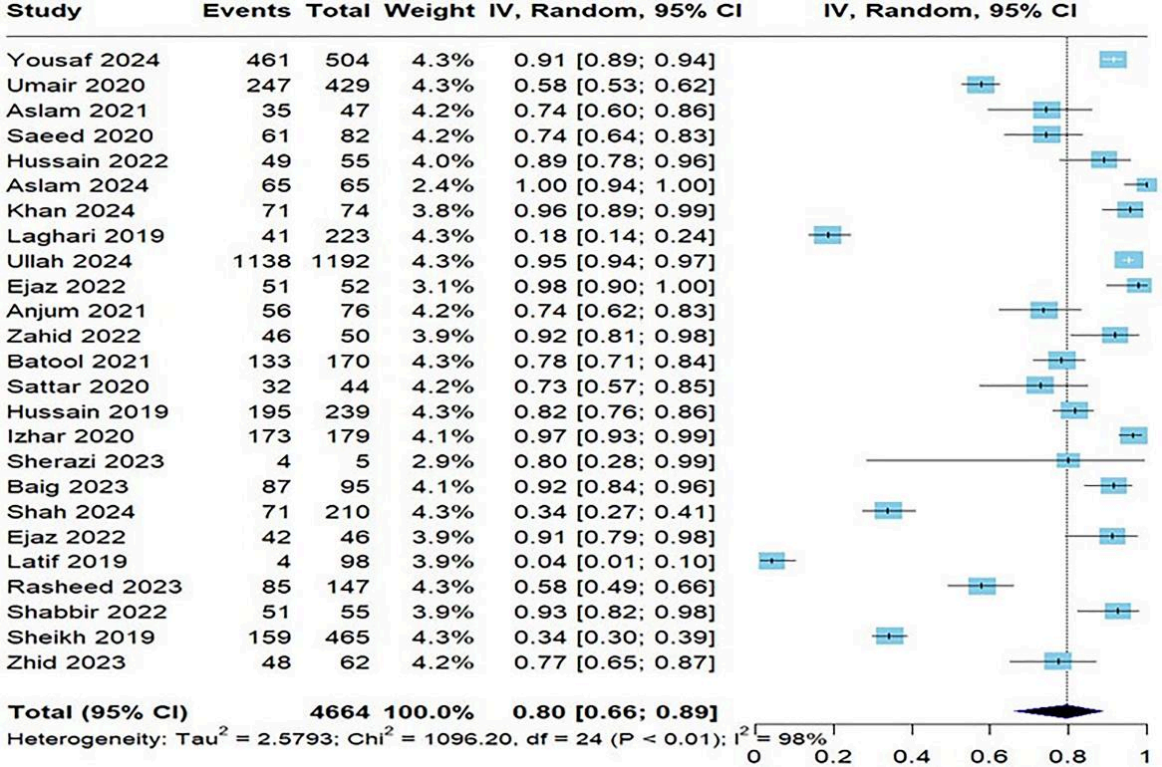
Forest plot illustrating the pooled prevalence resistance to ampicillin to *Salmonella typhi*.

**Figure 3.**
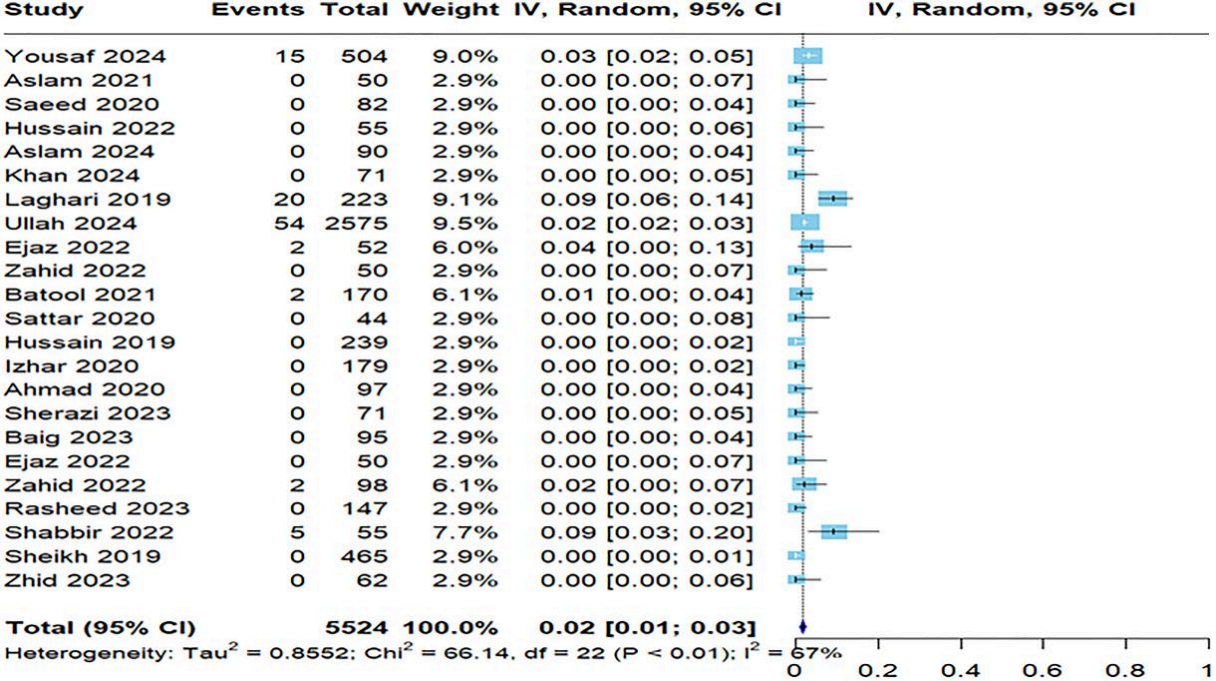
Forest plot illustrating the pooled prevalence resistance to meropenem to *Salmonella typhi*.

Table 2 provides a detailed summary of antibiotic resistance to *S typhi* in the 31 studies, showcasing the resistance rates for various antibiotics. Notably, the resistance was highest for nalidixic acid with a prevalence of 92% (95% CI, 88%–95%) across 3 studies (Supplementary Figure 1), followed by ampicillin with 25 studies reporting a resistance rate of 80% (95% CI, 66%–89%; Figure 2). The resistance to ciprofloxacin was also notable at 64% (95% CI, 48%–77%) in 28 studies (Supplementary Figure 2). On the lower end, imipenem (Supplementary Figure 3) and meropenem showed minimal resistance at 3% (95% CI, 1%–6%) and 2% (95% CI, 1%–3%; Figure 3), indicating their effectiveness against *S typhi.* Amikacin had a prevalence of 22% (95% CI, 7%–52%; Supplementary Figure 4). Azithromycin was more resistant than meropenem and imipenem with a prevalence of 7% (95% CI, 3%–16%; Supplementary Figure 5). Cefepime in 7 studies demonstrated a resistance of 53% (95% CI, 37%–69%; Supplementary Figure 6). Nine studies reported cefixime and ceftizoxime resistance of 40% (Supplementary Figure 7) and 46% (Supplementary Figure 8), respectively. Resistance to ceftriaxone (Supplementary Figure 9) was indicated in 41% (95% CI, 28%–55%) of cases, with similar resistance rates observed in *S paratyphi* at 34% (95% CI, 21%–47%). These findings align with the concerns raised by the Centers for Disease Control and Prevention regarding resistance to third-generation cephalosporins in travelers from Pakistan. Chloramphenicol (Supplementary Figure 10) had a resistance of 59% (95% CI, 40%–75%), and cotrimoxazole (Supplementary Figure 11) resistance was 58% (95% CI, 38%–75%).

**Table 2.** Resistance of Different Antibiotics to *Salmonella typhi*

Drug Name	No. of Studies	No. of Events	No. of Participants	Prevalence (95% CI), %	*I* ^2^ Value, %
Amikacin	5	437	2739	22 (7–52)	99
Ampicillin	25	3659	4664	80 (66–89)	98
Azithromycin	26	1395	6095	7 (3–16)	95
Cefepime	7	2437	3358	53 (37–69)	98
Cefixime	9	617	1809	40 (13–75)	98
Ceftizoxime	9	1934	3015	46 (16–89)	99
Ceftriaxone	26	2661	5572	41 (28–55)	98
Chloramphenicol	26	4167	6226	59 (40–75)	98
Ciprofloxacin	28	3478	6400	64 (48–77)	96
Cotrimoxazole	23	2013	3519	58 (38–75)	96
Imipenem	18	135	4680	3 (1–6)	89
Meropenem	23	100	5524	2 (1–3)	67
Nalidixic acid	3	549	596	92 (88–95)	0

### Prevalence of Resistance in *S paratyphi*

**Figure 4.**
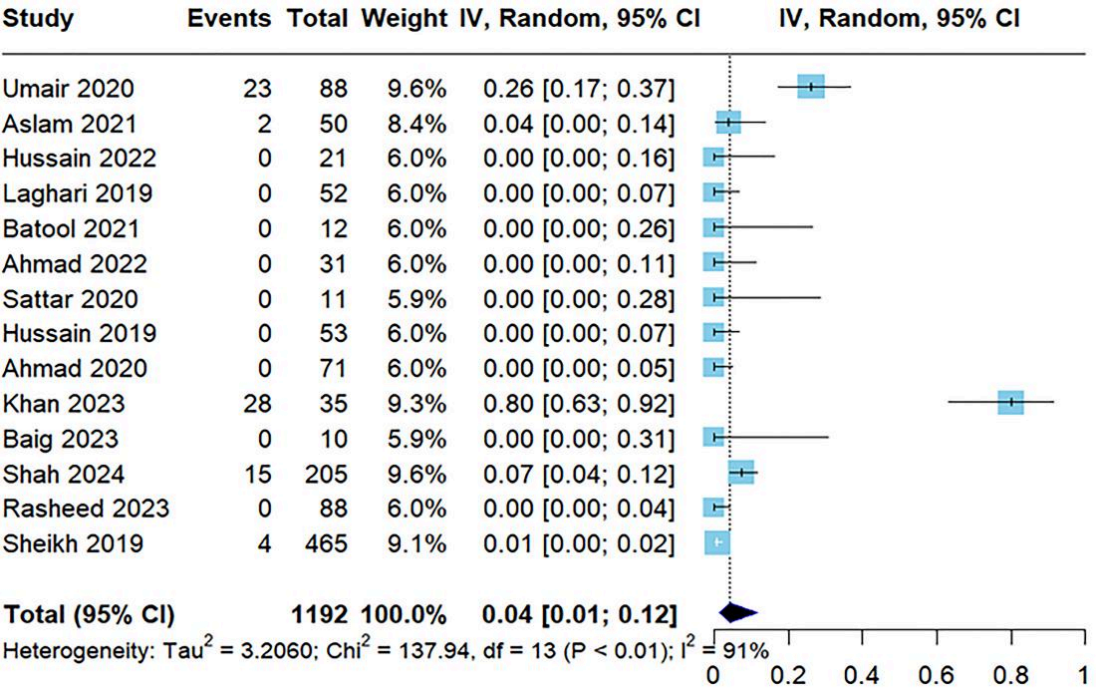
Forest plot illustrating the pooled prevalence resistance to azithromycin to *Salmonella paratyphi*.

**Figure 5.**
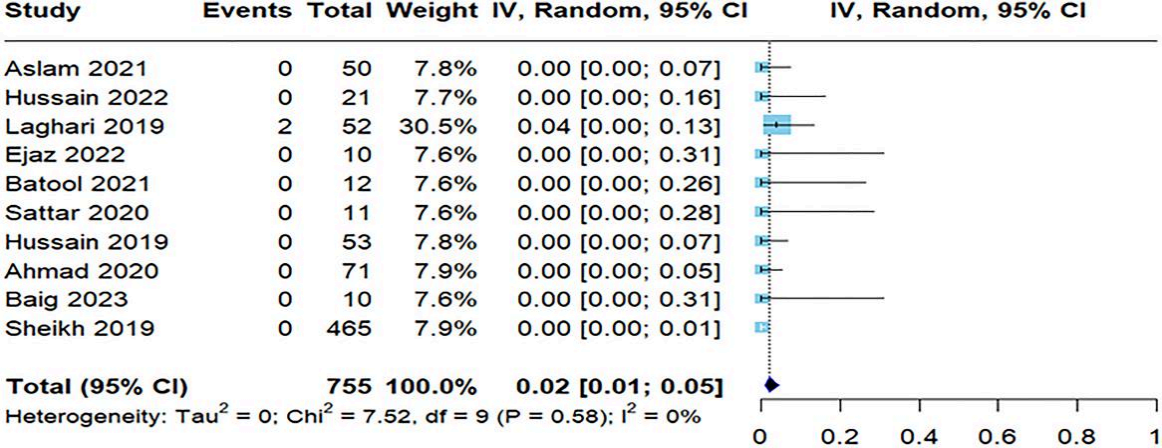
Forest plot illustrating the pooled prevalence resistance to meropenem to *Salmonella paratyphi*.

Table 3 presents the prevalence of antibiotic resistance to *S paratyphi* based on multiple studies. The antibiotics were evaluated across different numbers of studies with varying sample sizes. The prevalence of resistance is expressed as a percentage with a 95% CI, and the heterogeneity of the study results is quantified by the *I*^2^ value. A resistance prevalence of ampicillin was 34% (95% CI, 21%–50%) across 14 studies with a high degree of heterogeneity (*I*^2^ = 92%; Supplementary Figure 12). Azithromycin exhibited a lower resistance rate at 4% (95% CI, 1%–12%) from 14 studies with high heterogeneity (Figure 4). Cefepime and meropenem, tested in fewer studies, demonstrated a resistance prevalence of 50% (95% CI, 9%–91%) from 2 studies and 2% (95% CI, 1%–5%) from 10 studies, respectively, with meropenem showing minimal heterogeneity (Figure 5). Nalidixic acid (Supplementary Figure 13) was the most resistant drug with a resistance prevalence of 91% (95% CI, 82%–96%), and ciprofloxacin revealed a resistance of 51% (95% CI, 25%–77%) among 15 studies with the highest heterogeneity observed (*I*^2^ = 96%; Supplementary Figure 14).

**Table 3.** Resistance of Different Antibiotics to *Salmonella paratyphi*

Drug Name	No. of Studies	No. of Events	No. of Participants	Prevalence (95% CI), %	*I* ^2^ Value, %
Ampicillin	14	297	1142	34 (21–50)	92
Azithromycin	14	72	1192	4 (1–12)	91
Cefepime	2	30	60	50 (9–91)	0
Cefixime	5	25	769	4 (0–32)	85
Ceftizoxime	2	29	102	21 (0–1)	95
Ceftriaxone	12	48	1070	5 (2–15)	90
Chloramphenicol	16	188	1416	19 (8–36)	92
Ciprofloxacin	15	366	1324	51 (25–77)	96
Cotrimoxazole	14	251	1058	28 (13–51)	93
Imipenem	9	6	794	3 (1–6)	11
Meropenem	10	2	755	2 (1–5)	0
Nalidixic acid	3	174	190	91 (82–96)	0

### Prevalence of MDR and XDR

Table 4 summarizes the prevalence of MDR and XDR across the studies in this systematic review and meta-analysis. For *S typhi*, 19 studies yielded an MDR prevalence of 29% (95% CI, 19%–41%) with high heterogeneity (*I*^2^ = 96%), indicating substantial variations across studies (Supplementary Figure 15). The XDR prevalence was reported in 21 studies as 25% (95% CI, 12%–44%) with similarly high heterogeneity (*I*^2^ = 97%; Supplementary Figure 16). In 9 studies each, the prevalence of MDR and XDR for *S paratyphi* was notably lower. The prevalence of MDR was 9% (95% CI, 2%–28%) with an *I*^2^ value of 83%, suggesting considerable heterogeneity (Supplementary Figure 17), whereas the prevalence of XDR was 2% (95% CI, 1%–7%) with moderate heterogeneity (*I*^2^ = 48%; Supplementary Figure 18).

**Table 4.** Prevalence of Multidrug Resistance and Extensive Drug Resistance

	No. of Studies	No. of Events	No. of Participants	Prevalence (95% CI), %	*I* ^2^ Value, %
*Salmonella typhi*					
* *Multidrug resistance	19	1013	2744	29 (19–41)	96
* *Extensive drug resistance	21	596	2936	25 (12–44)	97
*Salmonella paratyphi*					
* *Multidrug resistance	9	32	755	9 (2–28)	83
* *Extensive drug resistance	9	2	740	2 (1–7)	48

### Subgroup Analysis

**Figure 6.**
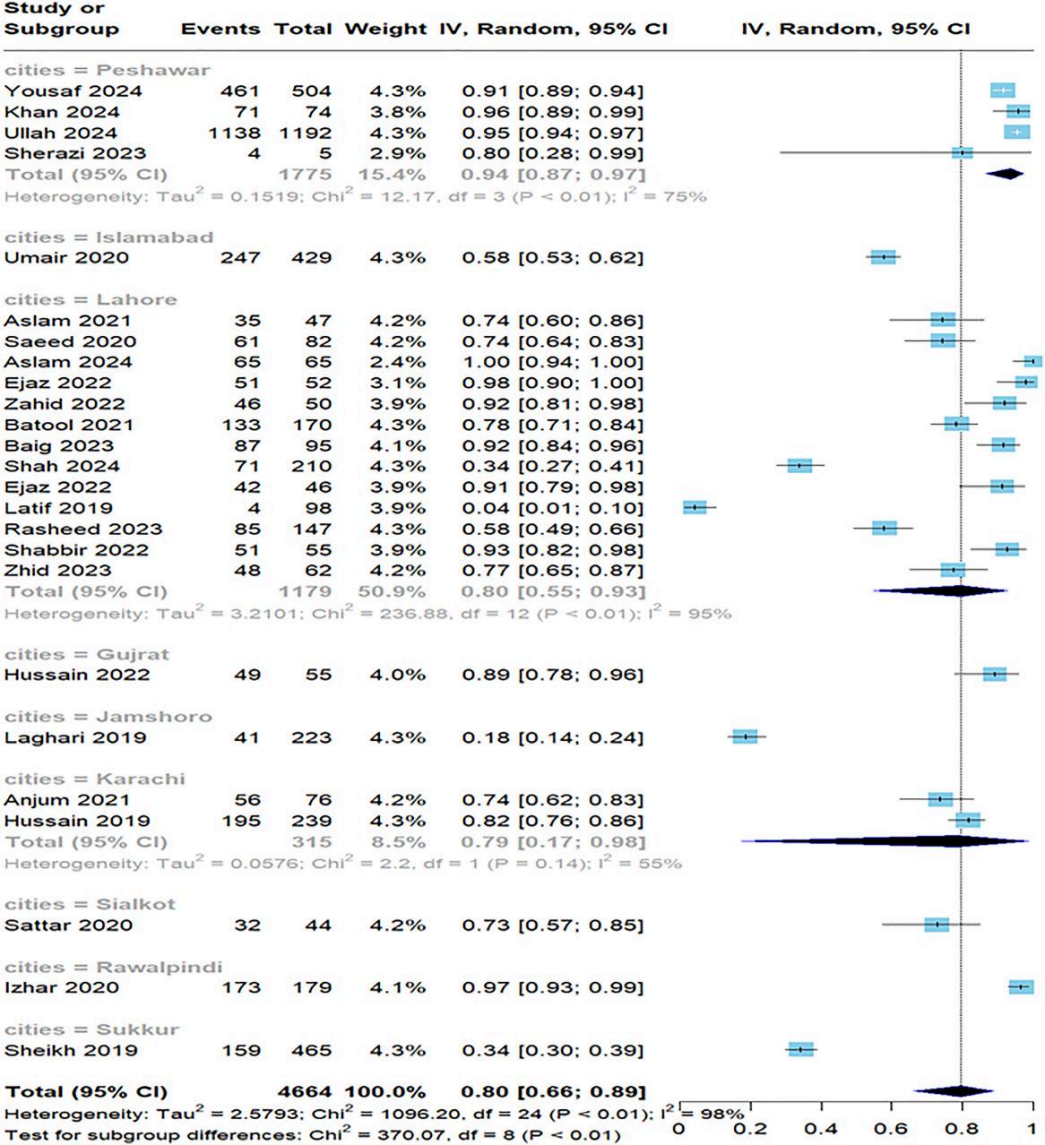
Forest plot illustrating the pooled prevalence of the subgroup based on cities.

**Figure 7.**
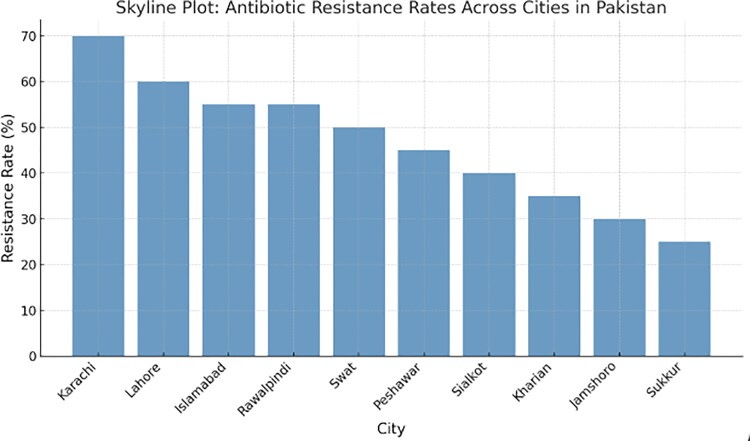
Skyline plot illustrating multidrug resistance across different cities in Pakistan.

In the subgroup analysis by city, the meta-analysis encompassed 4664 patients from various regions in Pakistan. The highest number of studies was conducted in Lahore (n = 14), showing a 52% pooled prevalence of antibiotic resistance. Furthermore, 3 and 4 studies were conducted in Islamabad and Peshawar, respectively, with Peshawar reporting a notably higher prevalence rate of 71%. Fewer studies were conducted from cities such as Karachi and Rawalpindi and smaller cities such as Sialkot and Sukkur, each adding singular studies to the analysis (Figure 6). The skyline plot shows resistance across geographic locations in Pakistan (Figure 7).

### Publication Bias

**Figure 8.**
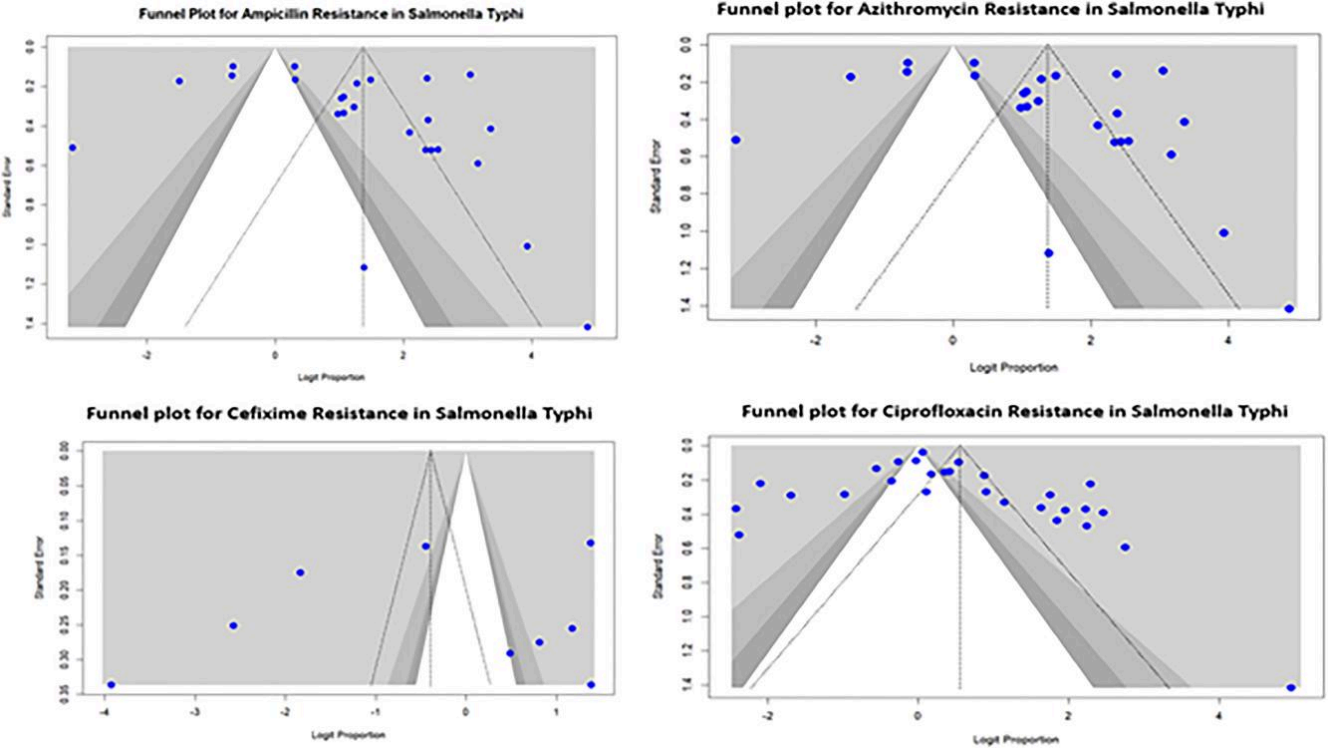
Funnel plots for detecting publication.

Funnel plots were created to assess the possibility of publication bias. The small asymmetry observed in funnel plots for ampicillin and azithromycin indicates the possibility of publication bias, whereby smaller studies with higher resistance rates are published more frequently. Conversely, the plots for cefixime and ciprofloxacin were more symmetrically distributed, indicating a decreased possibility of bias in these analyses (Figure 8). However, the existence of outliers, particularly in the ciprofloxacin plot, emphasizes the need for critical interpretation, as these may denote additional forms of bias, such as biased reporting in the published literature.

## DISCUSSION

Antibiotic resistance in *Salmonella* species represents a pressing public health challenge, particularly in countries such as Pakistan, where enteric fever, caused by *S typhi* and *S paratyphi*, remains endemic [44]. This systematic review and meta-analysis reviewed the existing data on antibiotic resistance among *Salmonella* strains across Pakistan over the past decade. The findings revealed an alarming prevalence of resistance to commonly used antibiotics.

As expected, our study revealed alarmingly high rates of AMR in *S typhi* and *S paratyphoid*, particularly in first-line antibiotics such as ciprofloxacin, nalidixic acid, and ampicillin. Although this finding is consistent with the growing body of literature on the increasing prevalence of resistant *Salmonella* strains, these resistance levels should be crucially quantified and reported for local and global health policy. The predictability of these trends underscores the urgent need for updated treatment guidelines and public health strategies reflecting the evolving nature of AMR in Pakistan. This study provides a clearer picture of regional variations in resistance, which can inform targeted interventions and antibiotic stewardship programs.

AMR has been recognized as a global health crisis, with projections suggesting that 1 person will die due to AMR pathogens every 3 seconds by 2050 [45]. The WHO has listed *Salmonella* as a priority pathogen for antibiotic resistance, highlighting its significant threat to global health [46]. Enteric fever, primarily caused by *S typhi* and *S paratyphi*, is a major public health concern in low- and middle-income countries, with South Asia bearing the highest burden [47]. Particularly, Pakistan has the third-highest typhoid rate globally and is among the top countries with the highest mortality rates from typhoid fever [48].

The findings of this review are consistent with the global trends of increasing AMR, particularly in resource-limited settings where health care infrastructure is frequently inadequate to address the challenges posed by resistant pathogens. The high resistance rates reflect a broader global trend where the misuse and overuse of antibiotics have led to the emergence of MDR and XDR strains, significantly complicating treatment options and leading to higher morbidity, mortality, and economic costs.

ESBL resistance has emerged as a significant concern in *S typhi* and *S paratyphi* strains in Pakistan. The mechanisms underlying ESBL production are largely mediated by chromosomes and plasmids. Interestingly, the spread of ESBL resistance significantly varies by location. For example, in regions such as Peshawar, the resistance rate for ESBL was notably higher (71%) when compared with Lahore (52%), which could be attributed to local health care infrastructure and antibiotic-prescribing practice differences. This regional variation highlights the need for localized treatment protocols to address ESBL resistance more effectively. This regional disparity in the resistance rates could be attributed to several factors: differences in health care practices, antibiotic-prescribing behaviors, and the availability of clean water and sanitation facilities. In regions such as Peshawar, where health care infrastructure may be less developed, antibiotics are more commonly overused and misused, resulting in higher resistance rates. Furthermore, the socioeconomic conditions in different regions play a crucial role in shaping antibiotic resistance patterns. In areas with limited health care access, self-medication and the empirical use of antibiotics without proper diagnostic confirmation are common practices, contributing to the development and spread of resistant strains [49]. The findings of this study emphasize the need for region-specific interventions and public health strategies to address antibiotic resistance more effectively. The high prevalence of resistance to first-line antibiotics, such as nalidixic acid, ampicillin, and ciprofloxacin, as observed in this study has significant implications for the treatment of enteric fever in Pakistan. The resistance rate of nalidixic acid, in particular, was highest at 92% for *S typhi* and 91% for *S paratyphi*, making it largely ineffective for empirical therapy in the region. Similarly, the high resistance rates to ciprofloxacin (64% for *S typhi* and 51% for *S paratyphi*) are concerning, as ciprofloxacin has been a key antibiotic for the treatment of enteric fever for many years. The emergence of the MDR and XDR strains of *Salmonella* complicates the treatment landscape. MDR *S typhi* was reported in 29% of cases, whereas XDR *S typhi* was found in 25% of cases. For *S paratyphi*, the prevalence of MDR and XDR was lower at 9% and 2%, respectively. The treatment options for XDR typhoid are limited, with carbapenems and azithromycin being the only currently available effective antibiotics. However, the increasing resistance to azithromycin, as observed in neighboring countries such as Bangladesh, elevates concerns about the long-term efficacy of this antibiotic.

Although this study reported a relatively low resistance rate to carbapenems and macrolides, the presence of such resistance remains concerning. These antibiotics are crucial for treating XDR strains, and any increase in resistance could severely limit treatment options, making it imperative to continue monitoring resistance to these last-resort therapies. Carbapenem resistance in *S typhi* and *S paratyphi* is typically mediated by plasmid-borne carbapenemase genes—namely, carbapenemase-producing carbapenem-resistant enterobacterales, although chromosomal mechanisms may also play a role. These mechanisms can significantly compromise treatment options, especially in managing XDR strains. The increasing production of carbapenemase-producing carbapenem-resistant enterobacterales in Pakistan highlights the urgent need for more targeted surveillance and novel treatment options to combat resistant infections.

These findings emphasize the need for updated clinical guidelines for the treatment of enteric fever in Pakistan. Currently, the standard treatment guidelines for typhoid fever recommend first-line antibiotics such as ciprofloxacin, ceftriaxone, and azithromycin. However, as revealed in our study, these antibiotics are increasingly ineffective due to their high resistance rates. Ciprofloxacin and ceftriaxone have shown alarming resistance rates of 64% and 41% in *S typhi*, respectively, rendering them suboptimal for empirical therapy in many regions. With the emergence of MDR and XDR strains, current guidelines should be revised to incorporate azithromycin and meropenem as frontline alternatives, especially in Peshawar and Karachi, where resistance is most prevalent.

Furthermore, regional variation in resistance patterns highlights the need for tailored treatment protocols. Clinicians should adjust antibiotic choices based on local resistance data, using ceftriaxone and ciprofloxacin primarily for susceptible cases. This problem was exacerbated by the widespread misuse and overuse of antibiotics in some areas, particularly due to empirical prescriptions without proper diagnostics. Therefore, antibiotic stewardship programs are essential to ensure the rational use of antibiotics and delay further spread of the resistant strains. The current reliance on older antibiotics, which have now become largely ineffective due to high resistance rates, must be reconsidered. Clinicians should be encouraged to choose antibiotics based on local resistance patterns and utilize newer antibiotics judiciously to prevent continued development of resistance.

Given the high prevalence of antibiotic resistance, vaccination has emerged as a crucial strategy to reduce the burden of enteric fever in Pakistan. Typhoid vaccines have been included on the WHO's list of essential medicines, reflecting the importance of immunization in controlling the spread of resistant strains. Vaccination not only reduces disease incidence but also decreases the reliance on antibiotics, thereby slowing the spread of resistant strains [50, 51]. Studies have shown that large-scale vaccination programs can significantly reduce the incidence of typhoid fever, especially in high-risk areas [52, 53]. However, vaccination coverage in Pakistan remains suboptimal, particularly in rural areas with limited health care access. Public health campaigns aimed at increasing awareness regarding the benefits of vaccination and improving vaccine coverage are essential for reducing enteric fever incidence and the associated antibiotic resistance burden. In addition, improving access to clean water and sanitation facilities, particularly in underserved regions, is critical for preventing the spread of *Salmonella* infections.

Although this systematic review and meta-analysis provide valuable insights into the prevalence of antibiotic resistance among *Salmonella* species in Pakistan, several limitations should be acknowledged. First, the heterogeneity among the studies was high. This heterogeneity could be due to differences in the study designs, sample sizes, geographic locations, and methods used for antibiotic susceptibility testing.

Subgroup analyses were conducted to explore the sources of heterogeneity; however, drawing definitive conclusions from such diverse data was challenging. Second, the studies in this review were predominantly observational, which may introduce biases related to the study design and data collection. Furthermore, the reliance on published data may have introduced publication bias, as studies reporting high resistance rates may be more likely published than those reporting lower rates. Funnel plots were used to assess publication bias; yet, the presence of bias cannot be entirely ruled out. Although this study is based on a convenience meta-analysis of published reports from 2014 to 2024, including studies based on stringent criteria ensures that the findings are relevant and reflect the most recent resistance trends. The time frame captures the critical period during the emergence of ESBL and XDR *S typhi* strains as significant threats in Pakistan, making this analysis particularly impactful in understanding the evolving landscape of AMR.

Future research should investigate the following several areas based on the study findings. First, more robust surveillance systems are required to monitor antibiotic resistance trends in Pakistan. Such systems should be integrated into the national health care infrastructure to provide real-time data on resistance patterns, which can inform treatment guidelines and public health interventions. Second, alternative treatment options should be developed for enteric fever, particularly in facing the increasing emergence of MDR and XDR *Salmonella* strains. The potential role of combination therapies, the use of newer antibiotics, and the exploration of nonantibiotic-based treatments (eg, phage therapy) should be investigated. Furthermore, future studies should explore the socioeconomic and behavioral factors contributing to the misuse of antibiotics in Pakistan. Understanding the underlying drivers of antibiotic overuse can inform targeted public health campaigns aimed at promoting the rational use of antibiotics. Long-term studies should also evaluate the impact of vaccination on reducing the incidence of enteric fever and the associated antibiotic resistance burden. Such research could provide valuable evidence to support the expansion of vaccination programs in Pakistan and other endemic regions.

## CONCLUSION

This review revealed an alarming prevalence of antibiotic-resistant *Salmonella* strains in Pakistan, particularly to commonly used antibiotics such as nalidixic acid, ampicillin, and ciprofloxacin. The emergence of MDR and XDR strains complicates the treatment of enteric fever, demonstrating the urgent revision of treatment guidelines and the implementation of public health strategies to combat antibiotic resistance. Vaccination, improved access to clean water and sanitation, and the rational use of antibiotics are critical components of the public health response to this increasing threat. Continued studies, policy interventions, and national and international cooperation are essential to safeguard public health and ensure the effective management of enteric fever in Pakistan and beyond.

Public health strategies should focus on expanding vaccination coverage, especially in rural areas, and improving diagnostic testing to ensure targeted treatments. Antibiotic stewardship programs are essential to reduce misuse and curb further resistance. Furthermore, improving access to clean water and sanitation will help reduce enteric fever transmission.

Overall, antibiotic resistance and effective control of enteric fever can be managed and ensured by updating treatment guidelines, enhancing vaccination efforts, and strengthening public health infrastructure.

## Supplementary Material

ofaf131_Supplementary_Data

## Supplementary Data

Supplementary materials are available at *Open Forum Infectious Diseases* online. Consisting of data provided by the authors to benefit the reader, the posted materials are not copyedited and are the sole responsibility of the authors, so questions or comments should be addressed to the corresponding author.

## Notes

***Financial support.*** This study, Prevalence of Antibiotic Resistance of Salmonella Species in Pakistan: A Systematic Review and Meta-Analysis, was conducted without any external funding. The authors declare that no financial support, grants, or funding from public, commercial, or nonprofit organizations was received for the research, authorship, or publication of this manuscript.

***Potential conflicts of interest*.** The authors affirm no conflicts of interest associated with the research, authorship, or publication of this study. None of the authors have personal or financial relationships with individuals, organizations, or entities that could influence the manuscript content or interpretation. Furthermore, there are no competing interests, whether professional, political, or financial, which may affect the credibility or integrity of the work.
