# Supplementary material for: Prevalence of the Antibiotic Resistance of Salmonella typhi and Salmonella paratyphi in Pakistan: A Systematic Review and Meta-analysis
Source: Open Forum Infect Dis. 2025 Mar 11;12(4):ofaf131. doi: 10.1093/ofid/ofaf131 (PMC11962718; doi:10.1093/ofid/ofaf131)
Supplement: ofaf131_Supplementary_Data [file ofaf131_supplementary_data.docx]

**SUPPLEMENTARY MATERIALS**

**
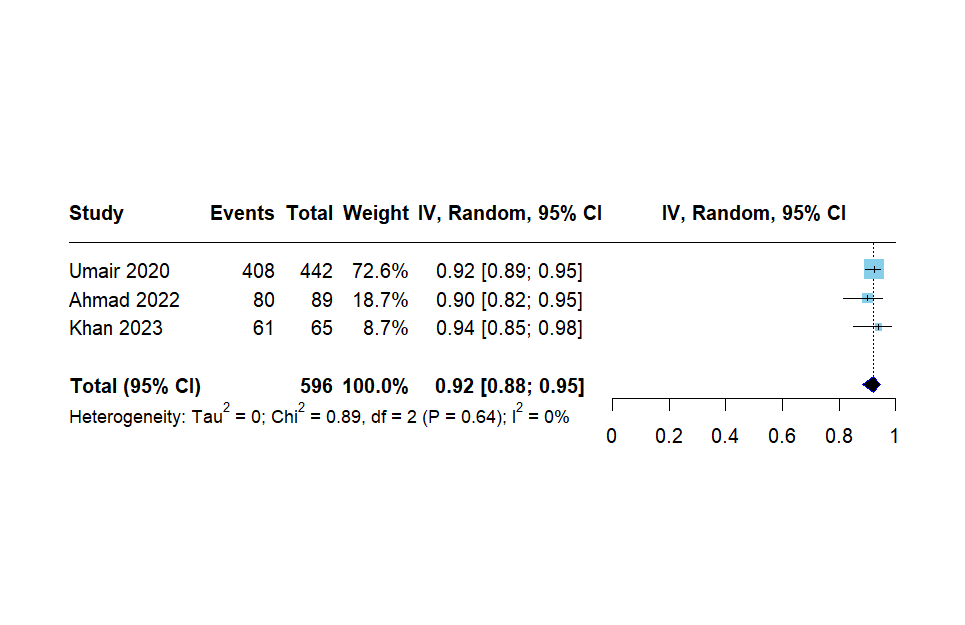
**

***FigureS1.*** *Forest plot illustrating pooled prevalence resistance of Nalidixic Acid to Salmonella typhi*

**
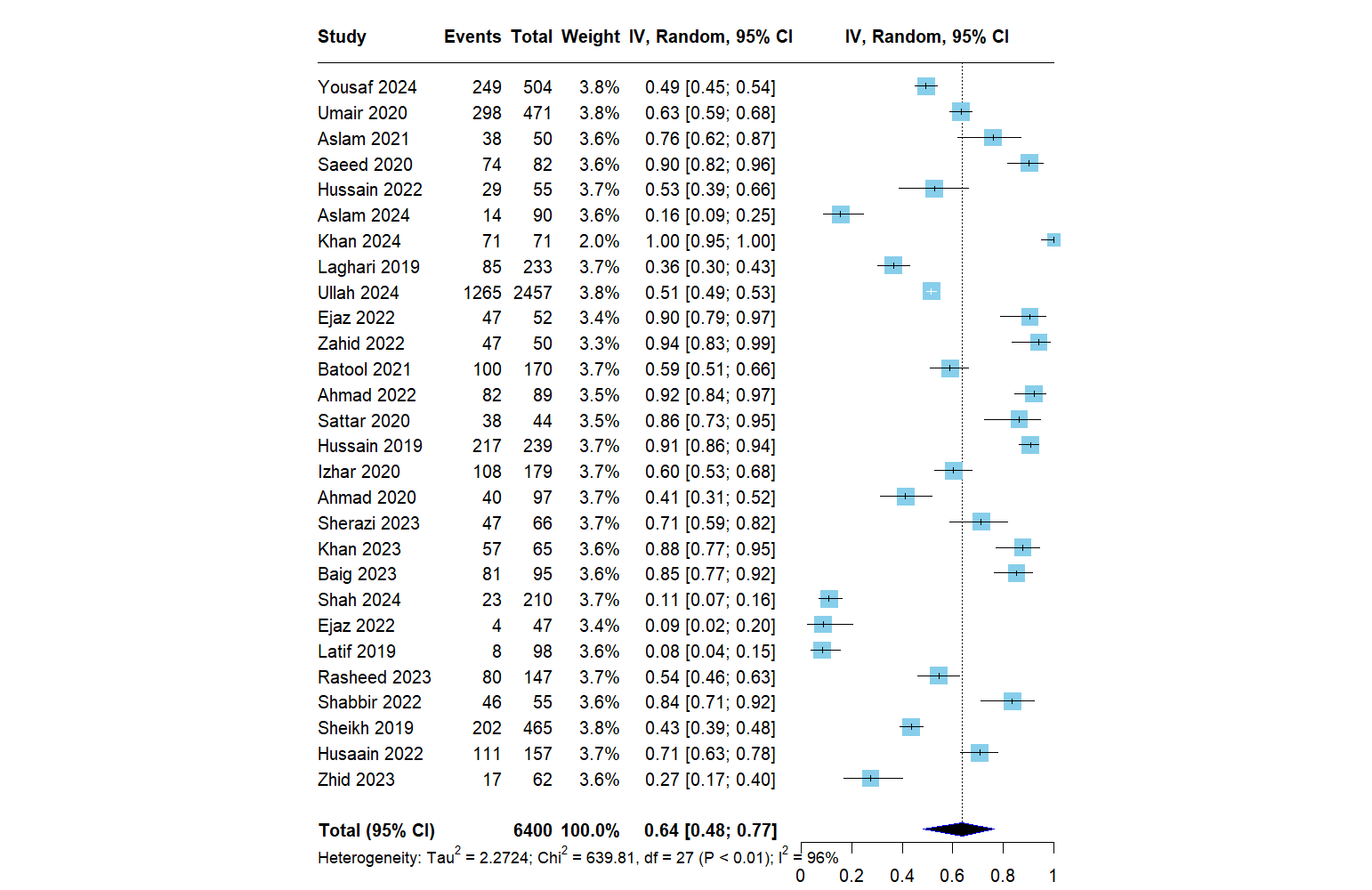
**

**Figure S2.** Forest plot illustrating pooled resistance of ciprofloxacin to *Salmonella Typhi*


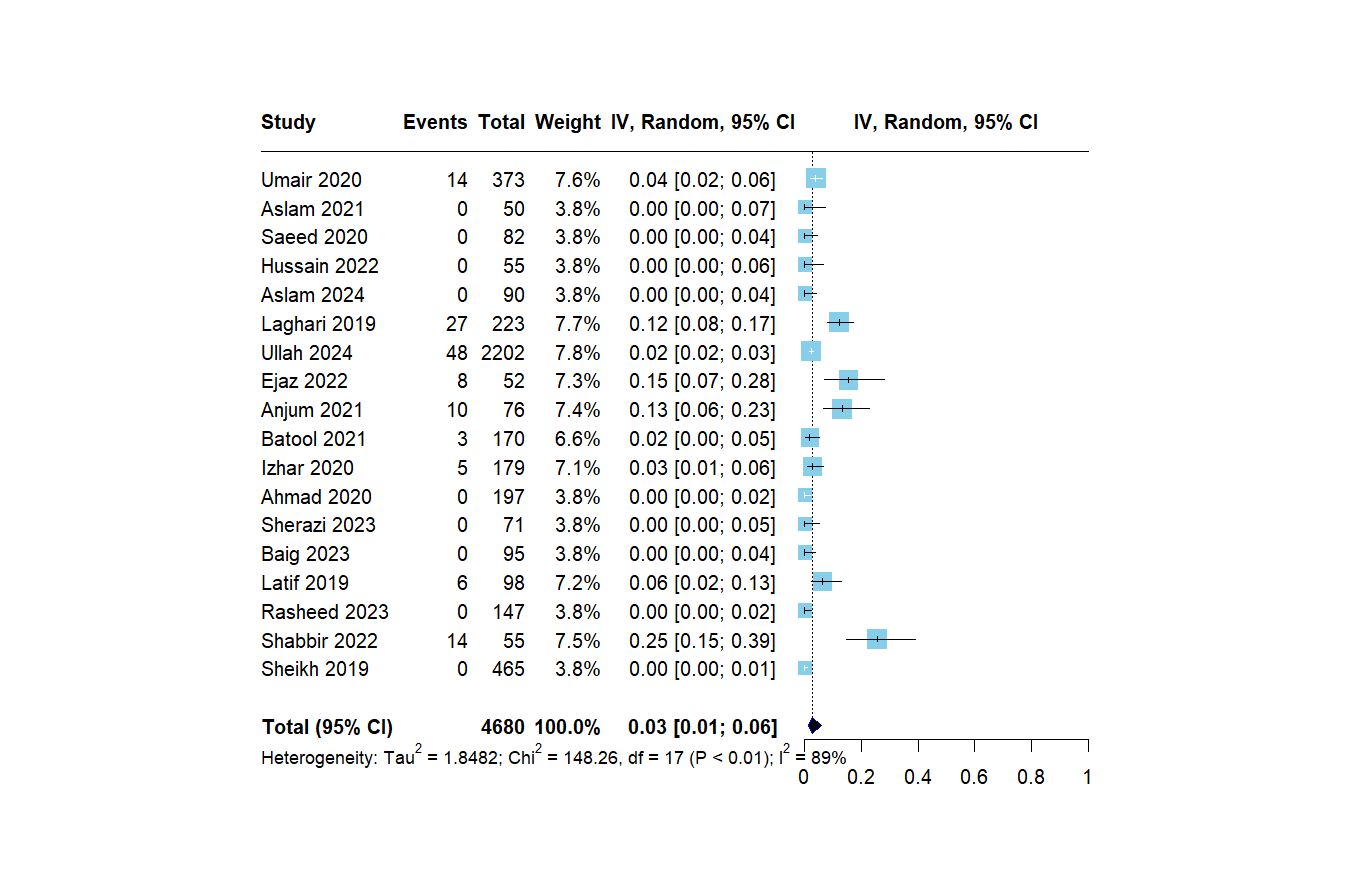


**Figure S3.** Forest plot depicting pooled resistance of Imipenem to *Salmonella Typhi*


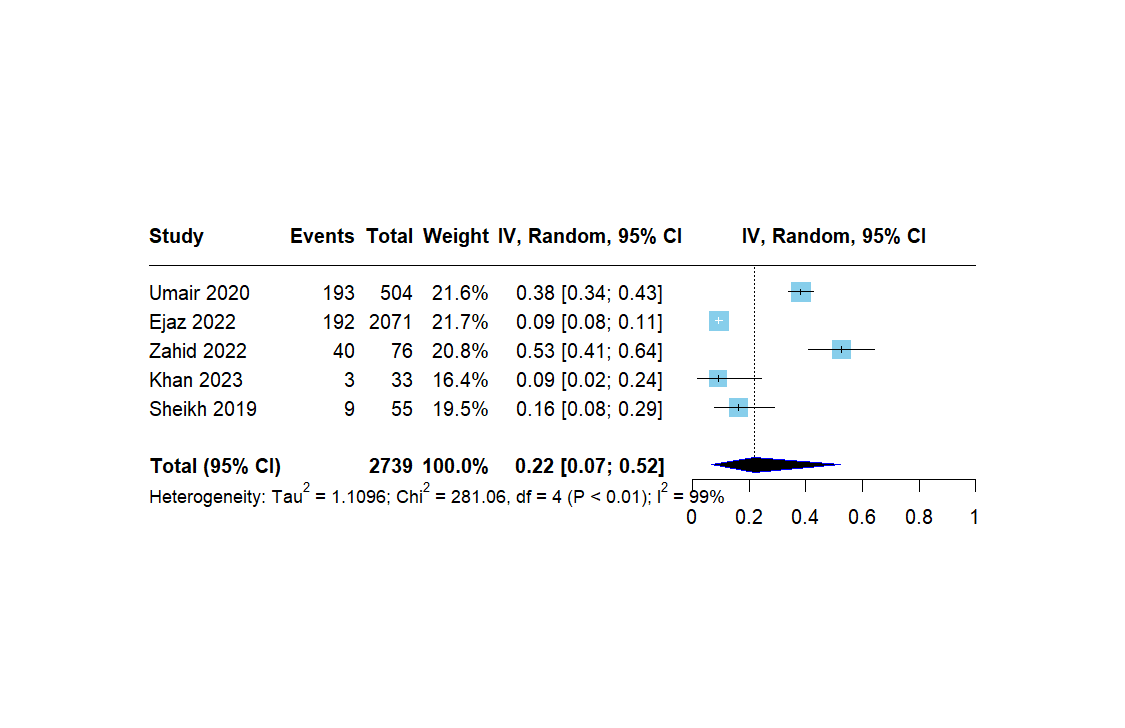


**Figure S4.** Forest plot depicting pooled resistance of Amikacin to *Salmonella Typhi*
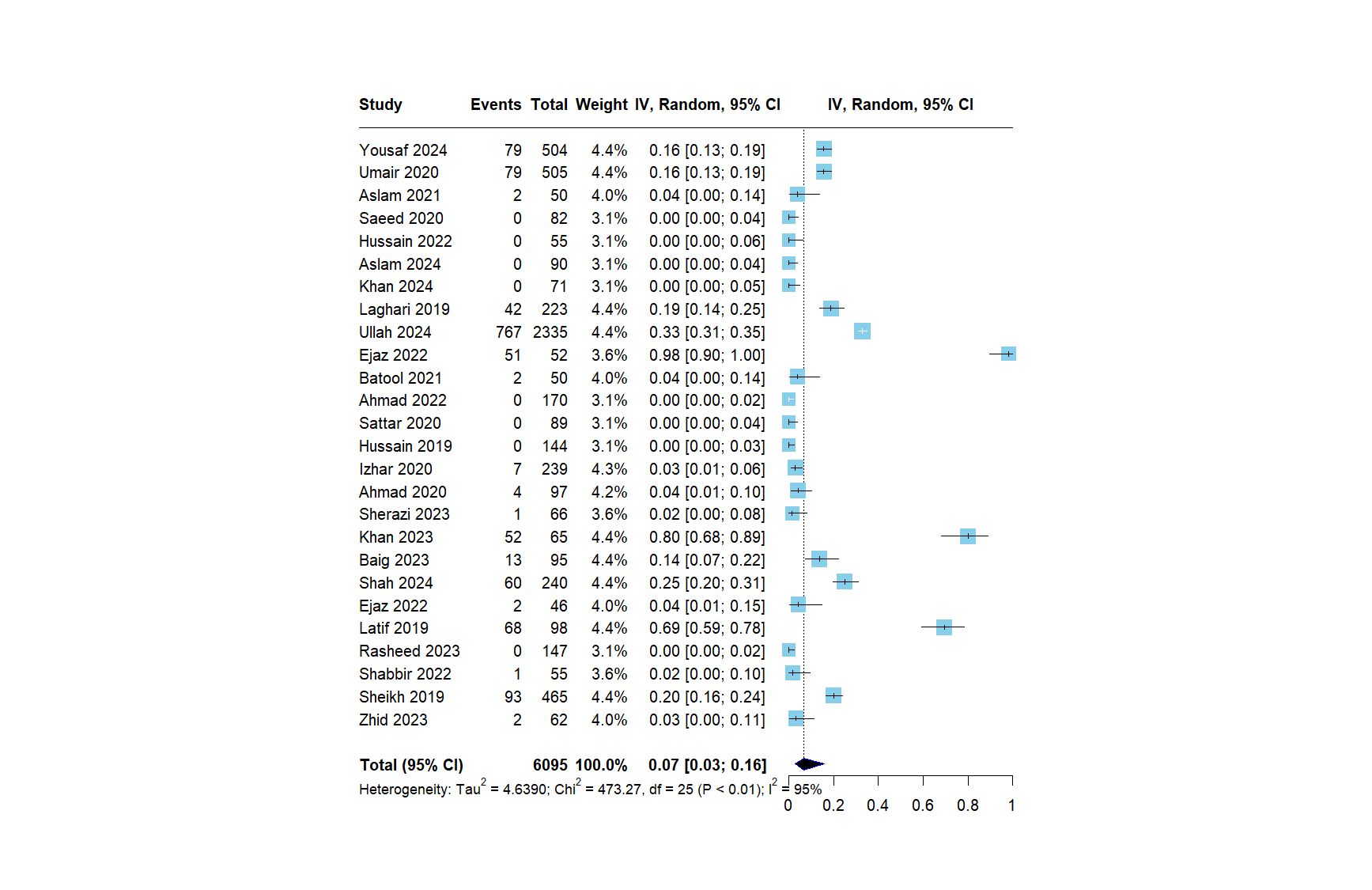


**Figure S5.** Forest plot depicting pooled resistance of Azithromycin to *Salmonella Typhi*


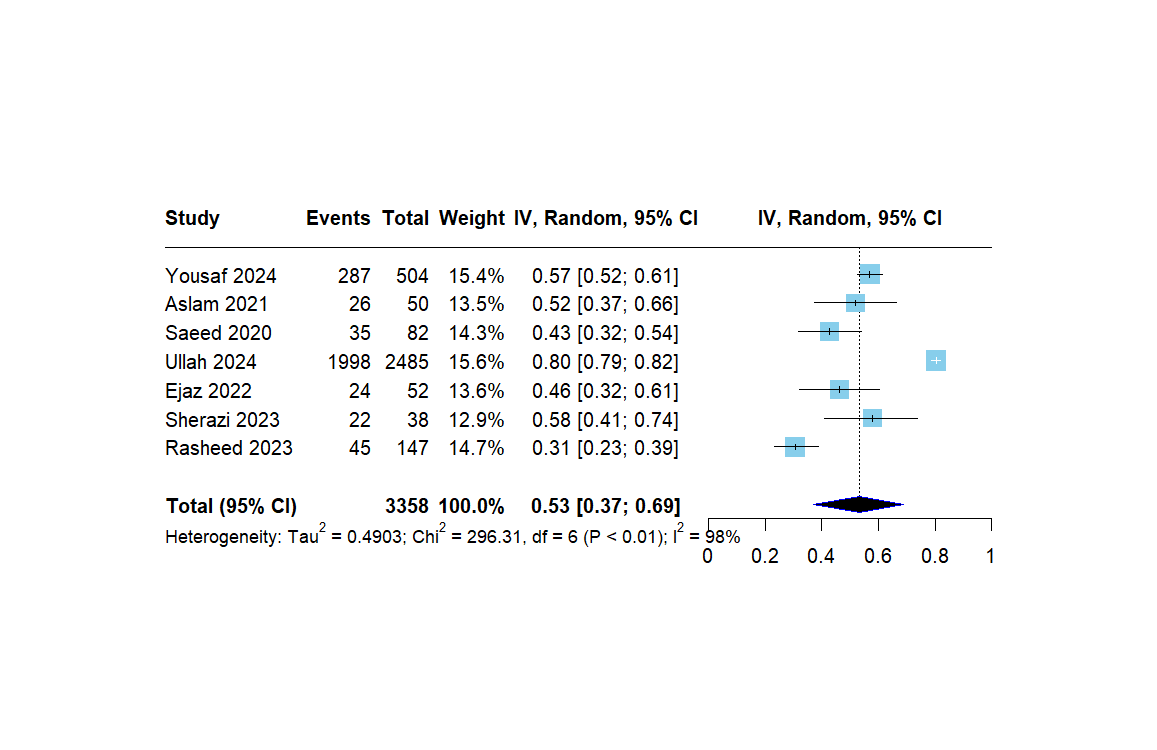
 **Figure S6.** Forest plot depicting pooled resistance of Cefepime to *Salmonella Typhi*


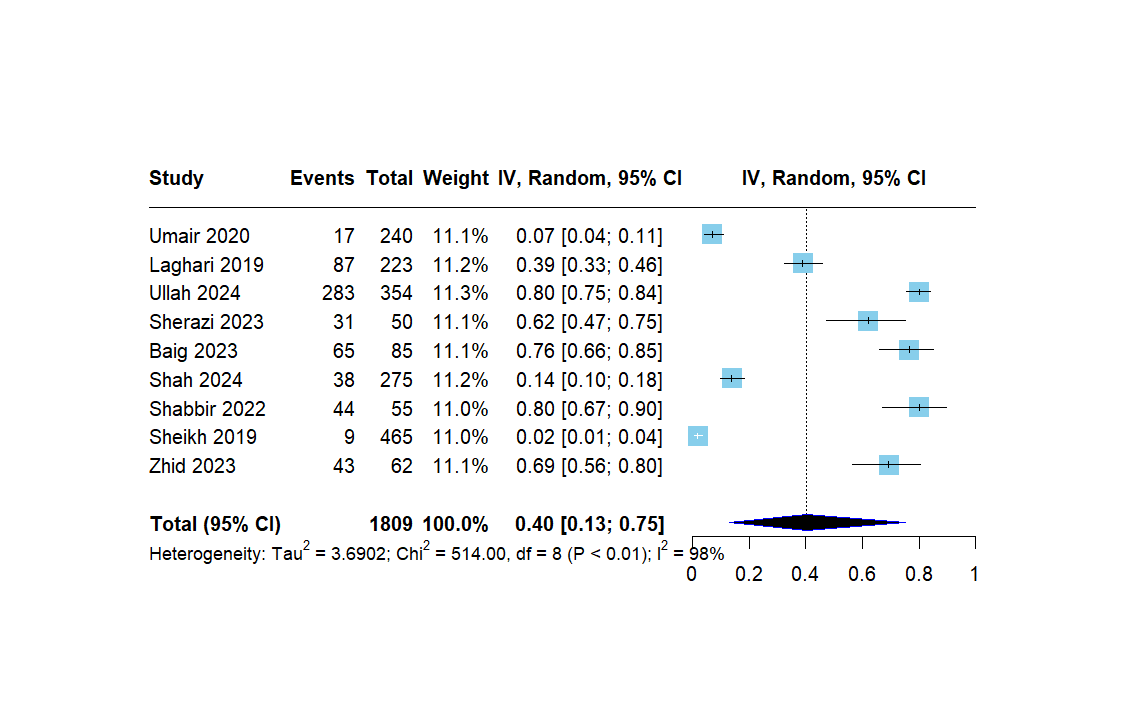


**Figure S7.** Forest plot depicting pooled resistance of Cefixime to *Salmonella Typhi*

**
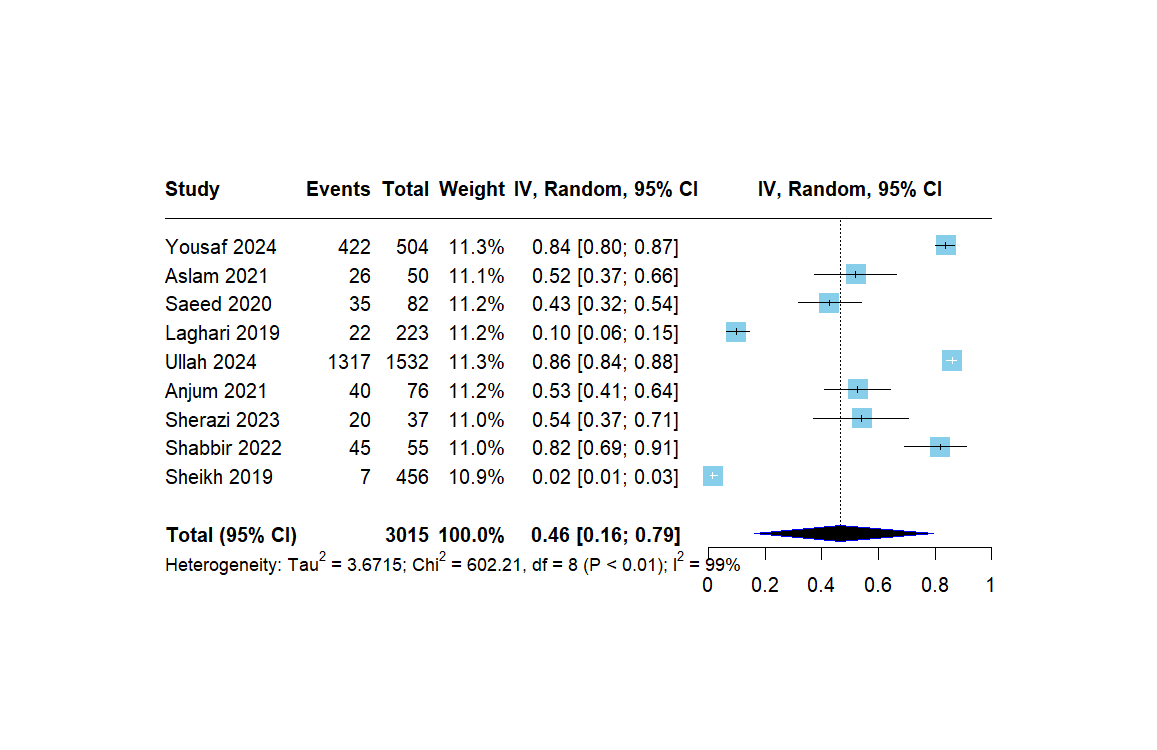
**

**Figure S8.** Forest plot depicting pooled resistance of ceftizoxime to *Salmonella Typhi*

**
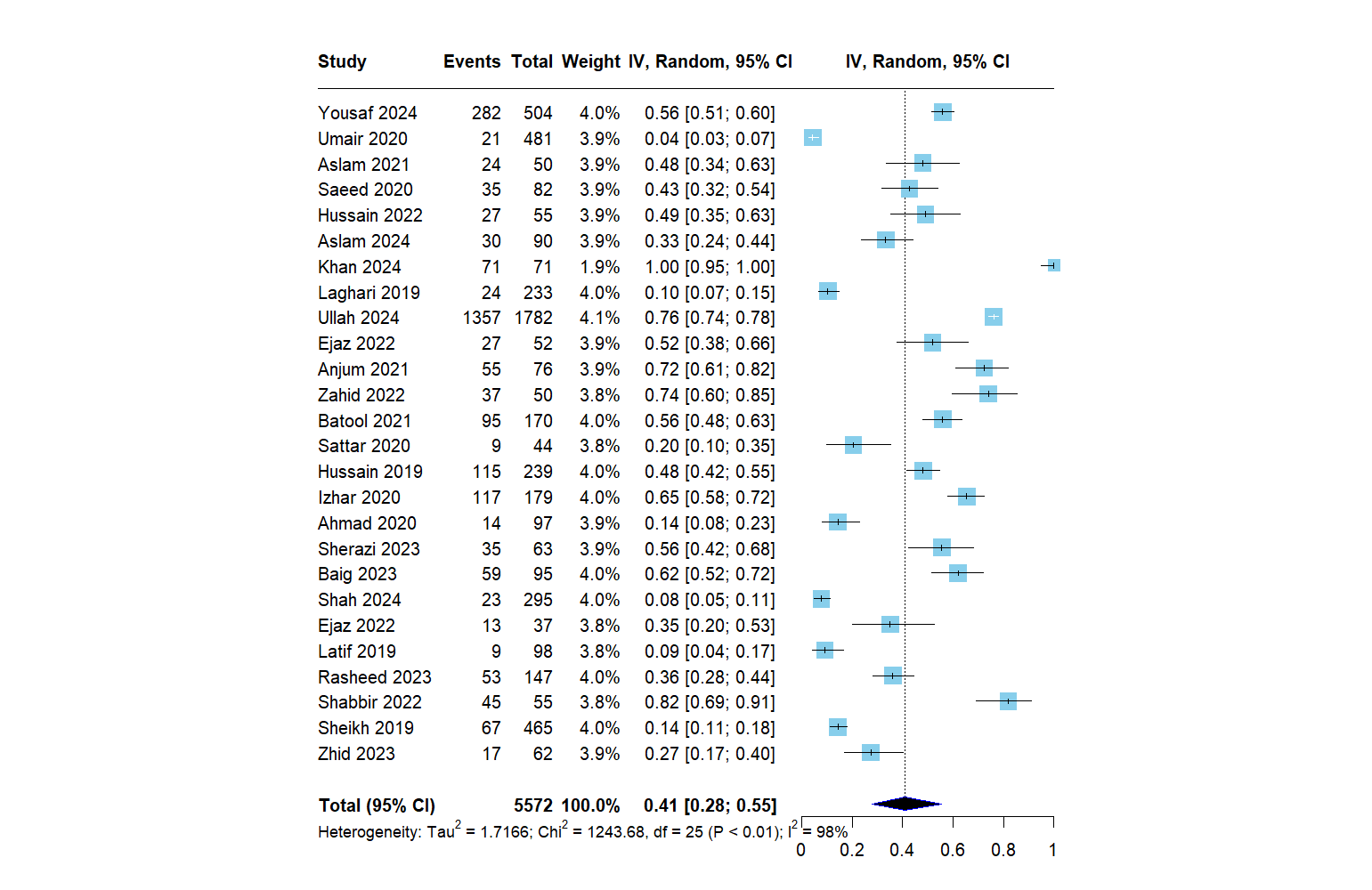
**

**Figure S9.** Forest plot depicting pooled resistance of ceftriaxone to *Salmonella Typhi*

**
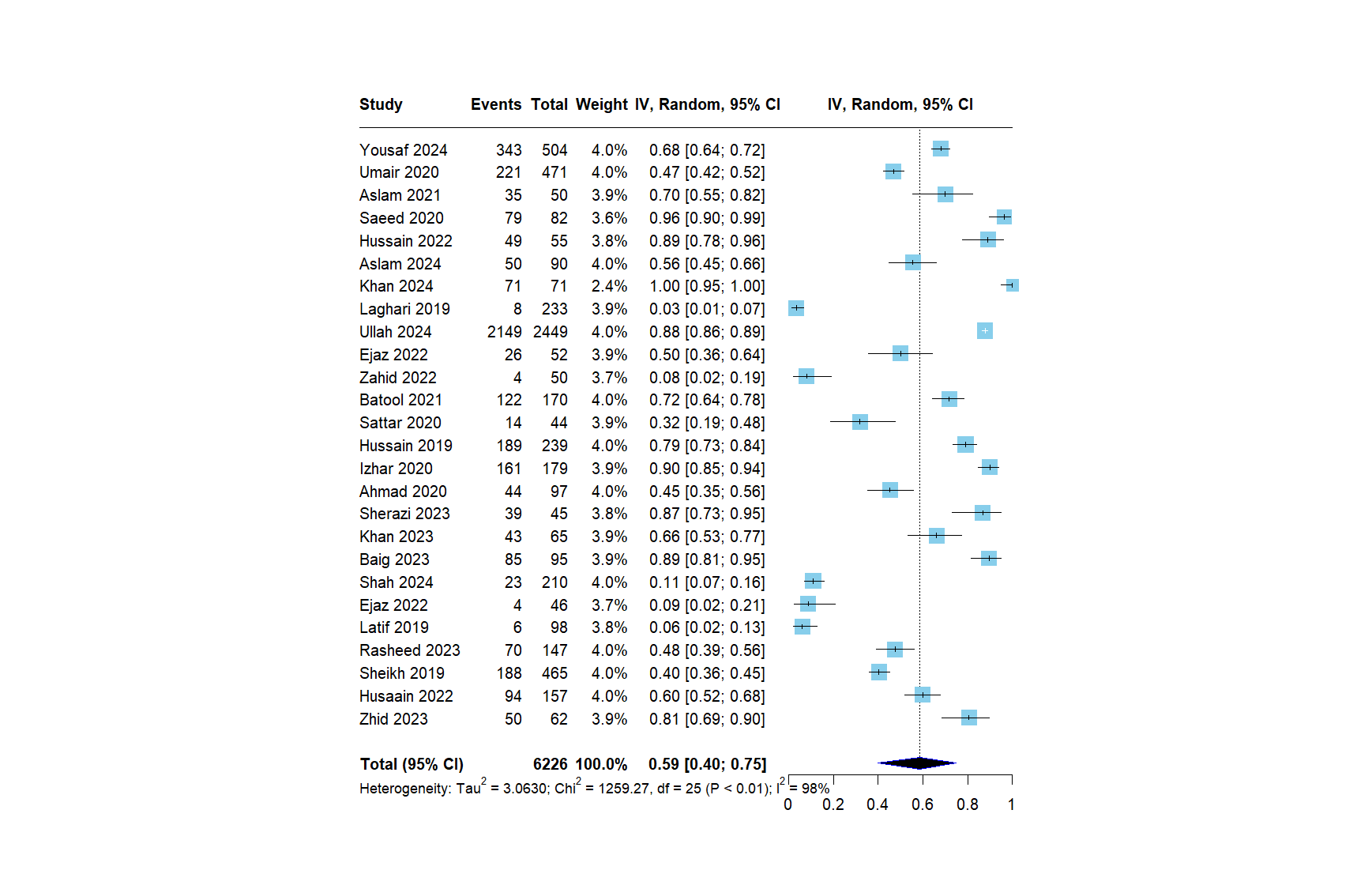
**

**Figure S10.** Forest plot depicting pooled resistance of Chloramphenicol to *Salmonella Typhi*

**
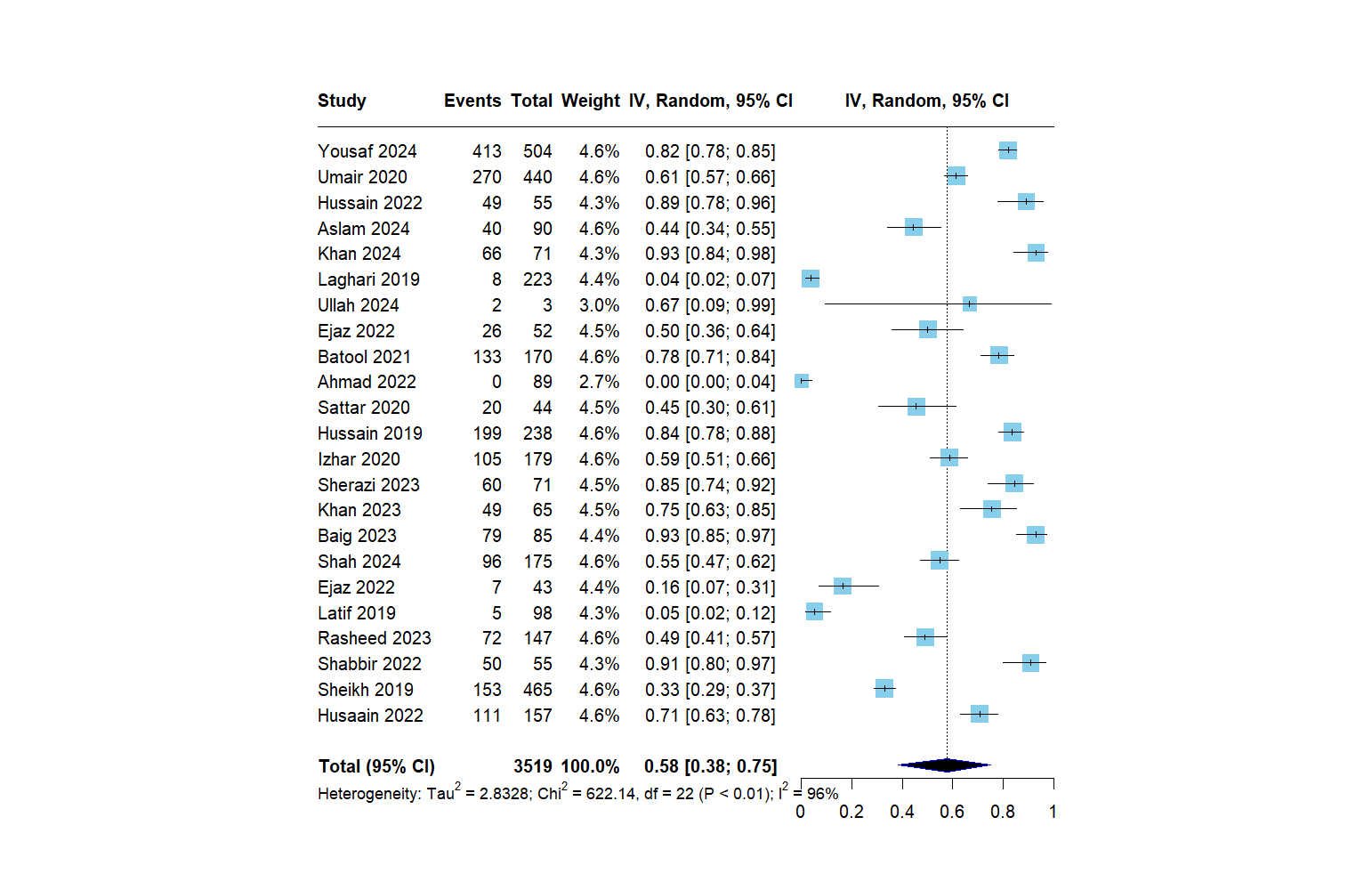
**

**Figure S11.** Forest plot depicting pooled resistance of Cotrimoxazole to *Salmonella Typhi*


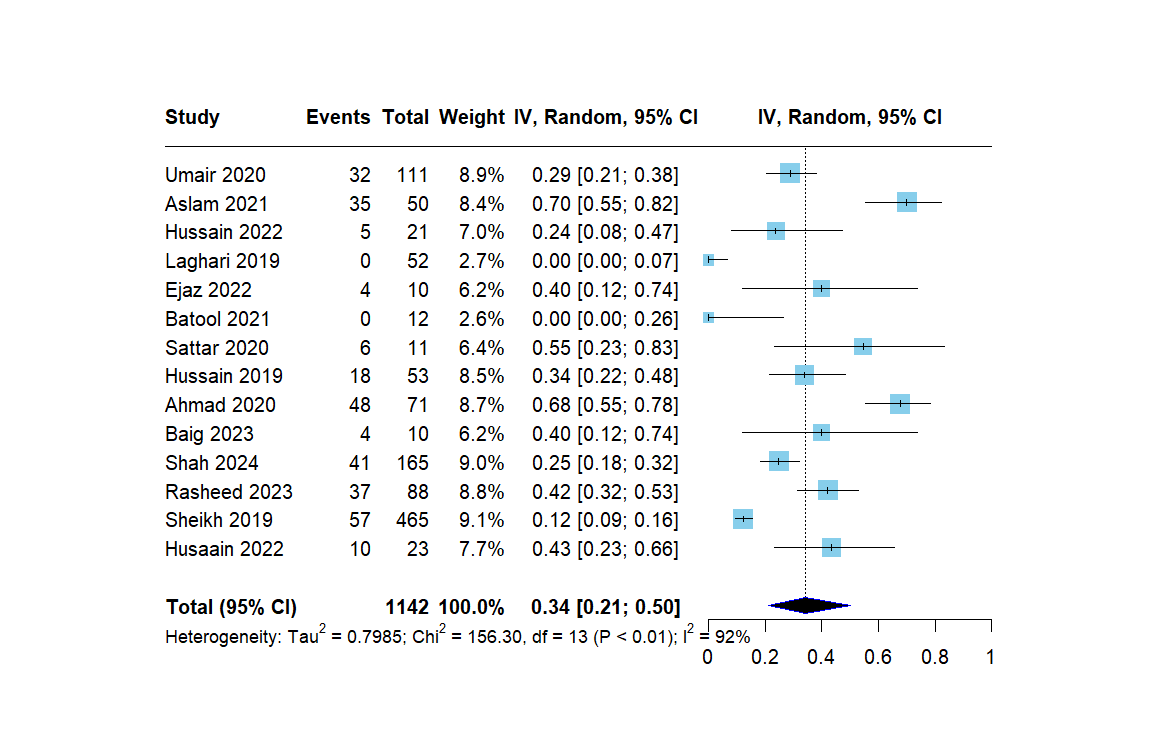


**Figure S12** Forest plot illustrating pooled prevalence resistance of Ampicillin to *Salmonella Para typhi*


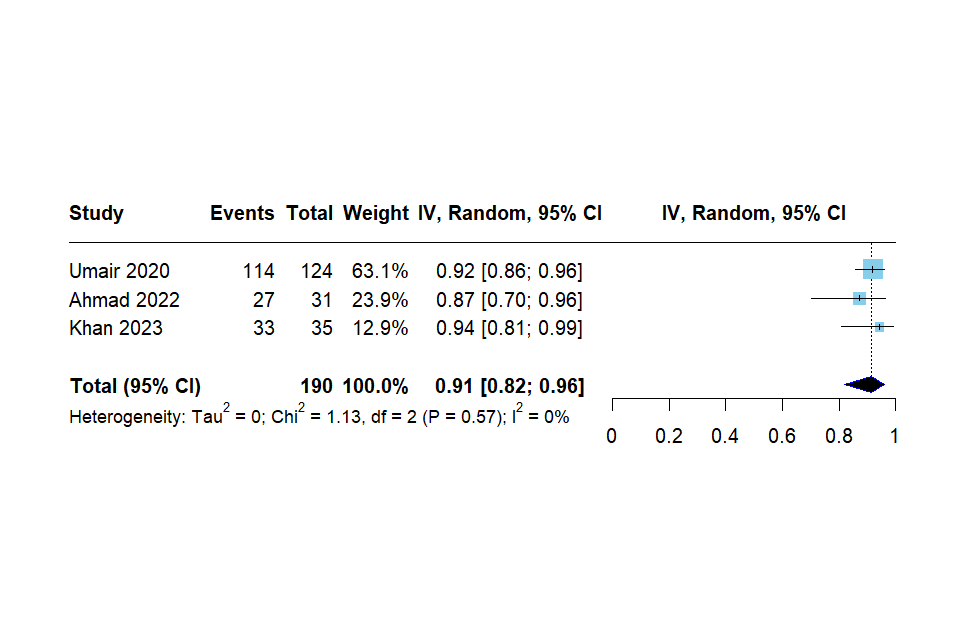


**Figure-S13** Forest plot illustrating pooled prevalence resistance of Nalidixic acid to Salmonella Para typhi


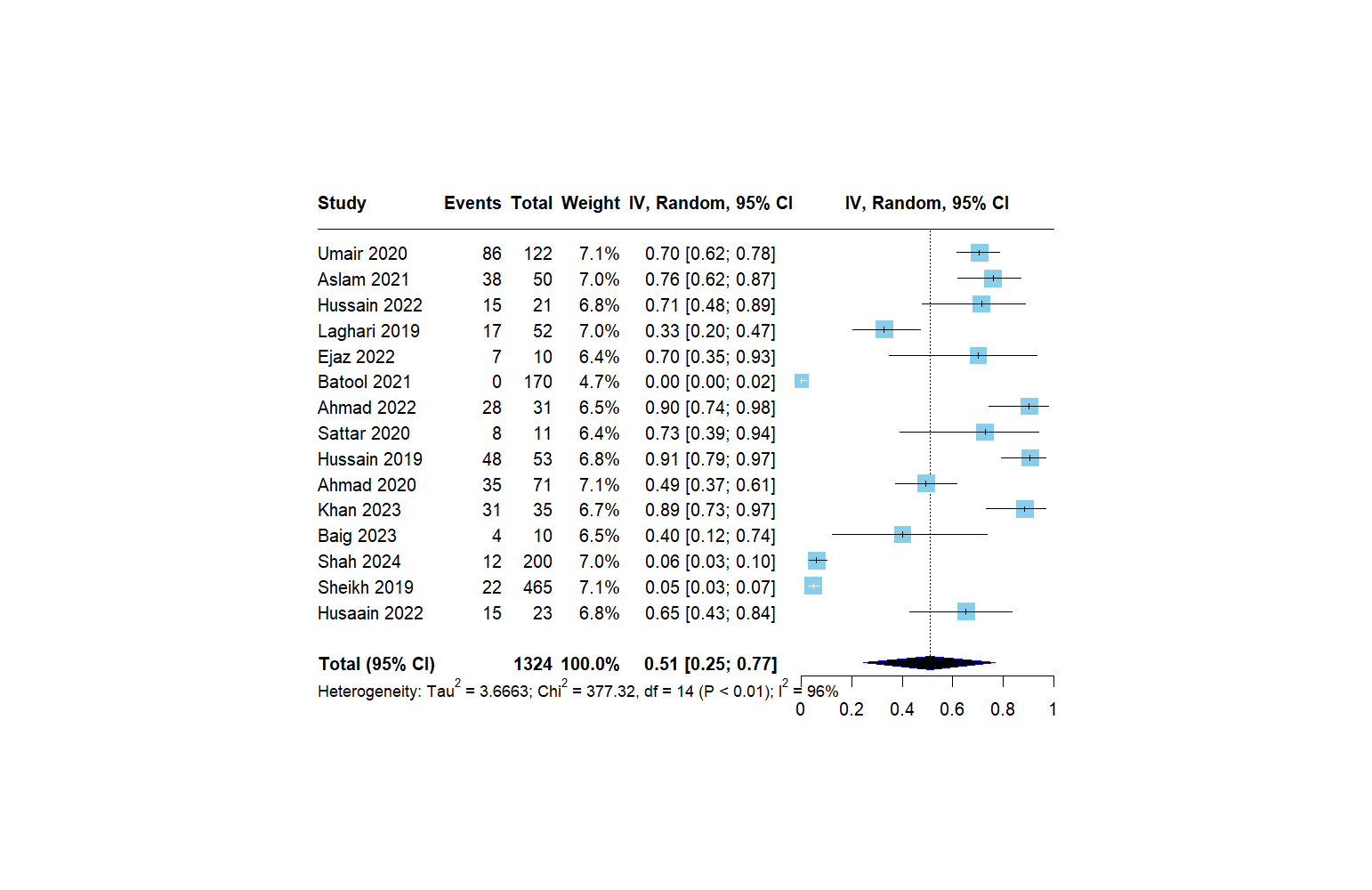


**Figure-S14** Forest plot illustrating pooled prevalence resistance of Ciprofloxacin to *Salmonella Para typhi.*

**
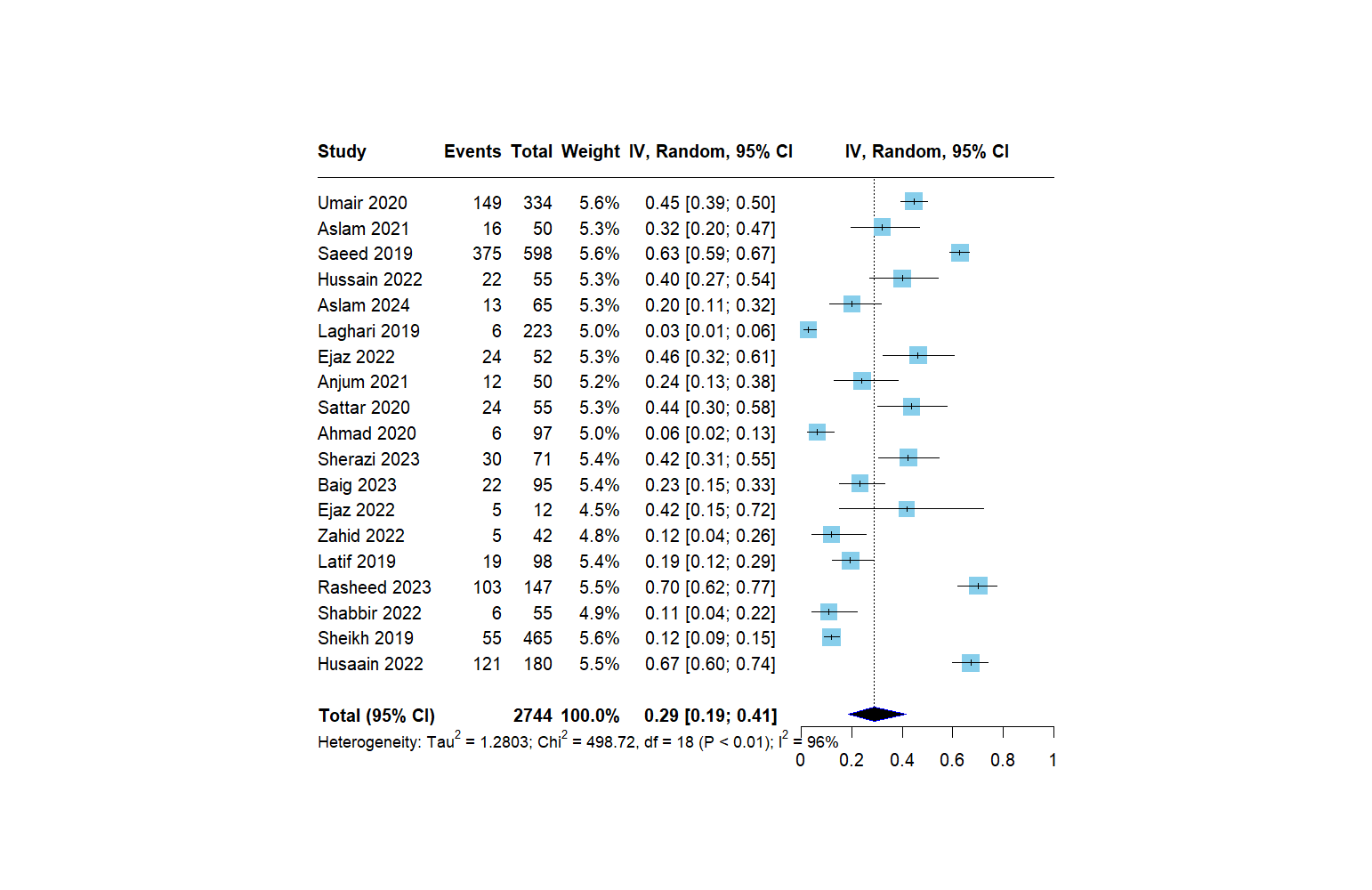
**

**Fig-S15** Forest plot illustrating the pooled prevalence of MDR to salmonella typhi

**
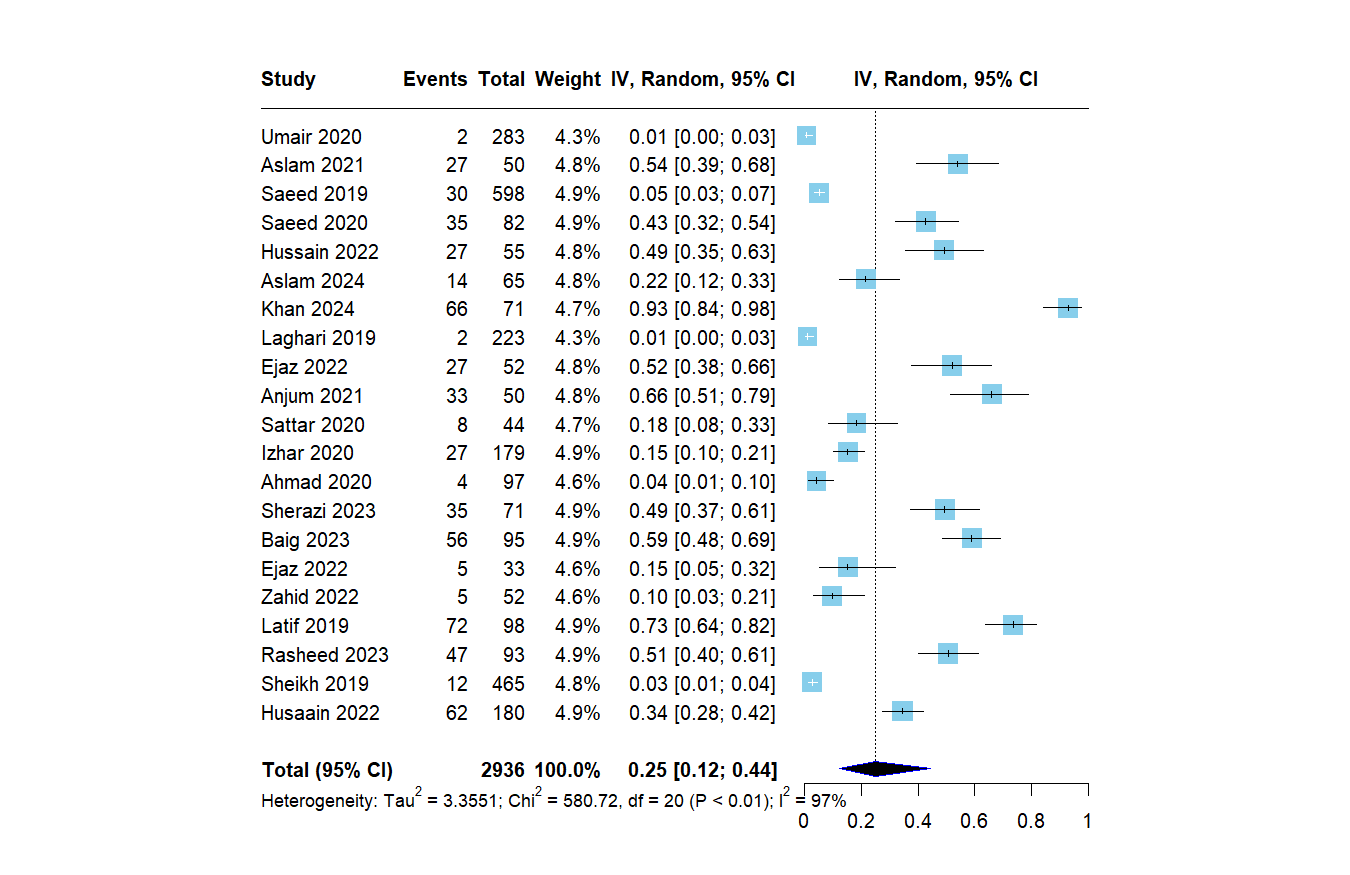
**

**Fig-S16** Forest plot illustrating the pooled prevalence of XDR to salmonella typhi

**
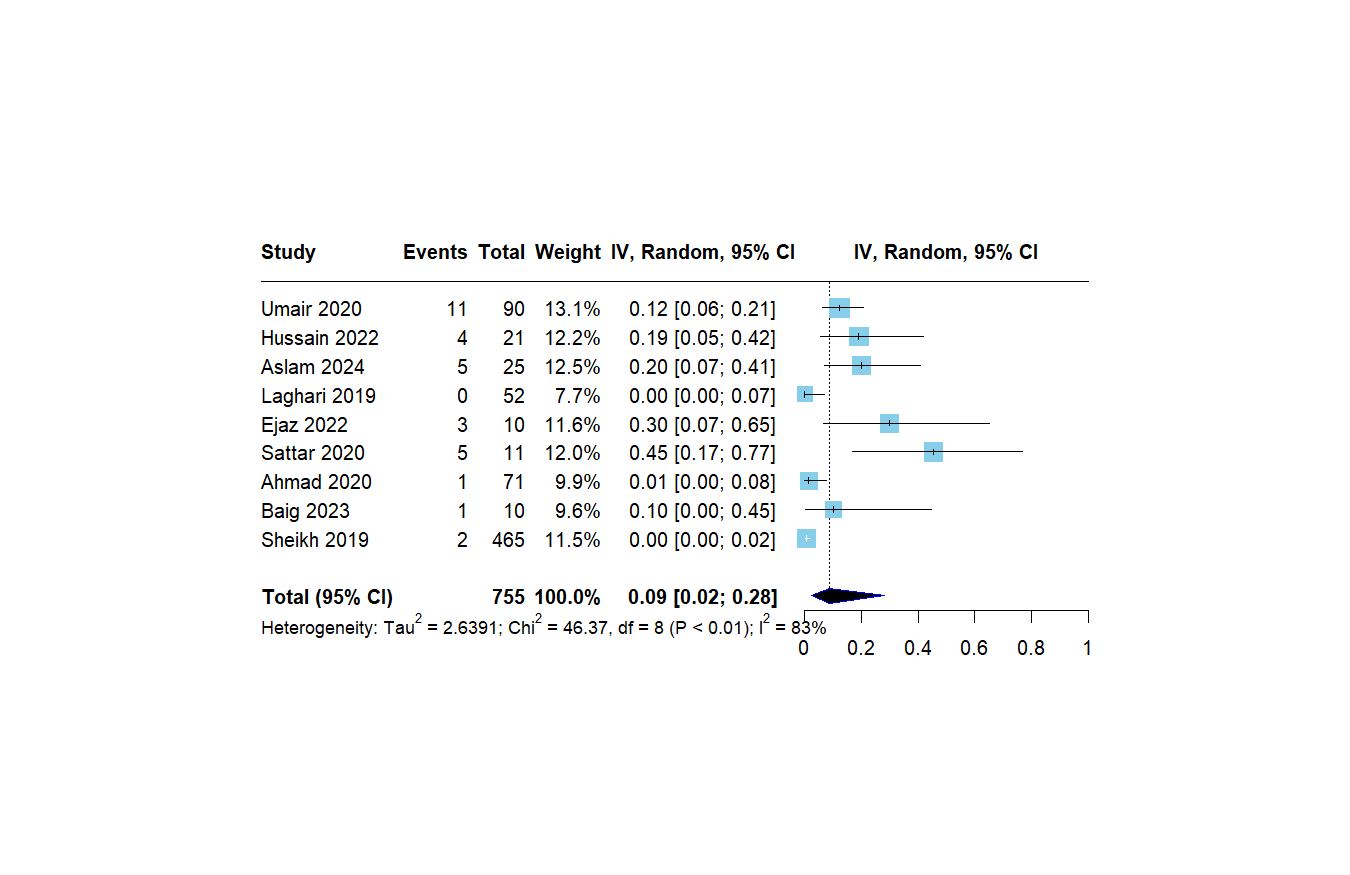
**

**Fig-S17** Forest plot illustrating the pooled prevalence of MDR to Salmonella Paratyphi

**
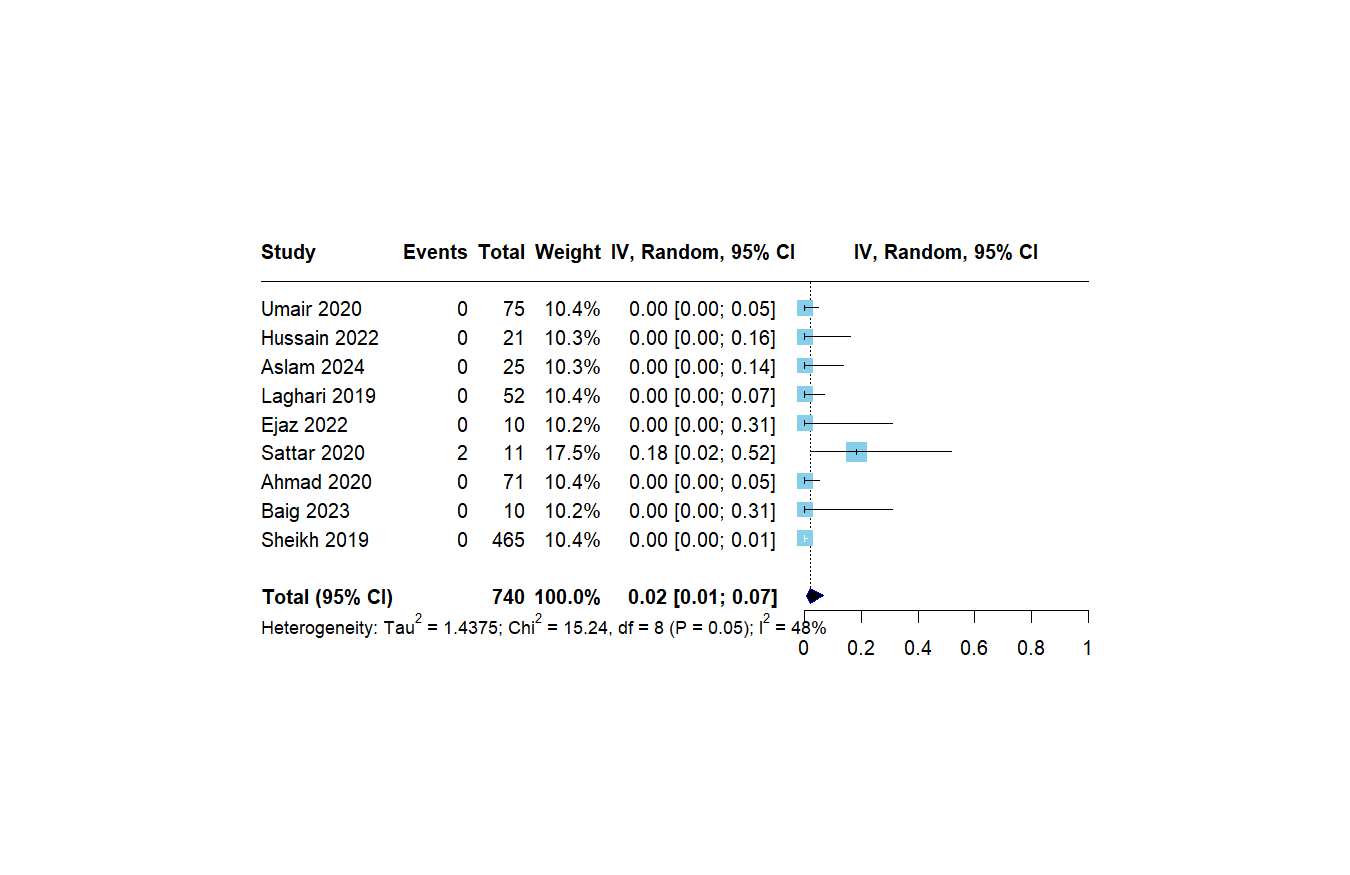
**

**Fig-S18** Forest plot illustrating the pooled prevalence of XDR to Salmonella Paratyphi

## **Table S1.** PRISMA Checklist

| **Section and Topic** | **Item #** | **Checklist item** | **Location where item is reported** |
| --- | --- | --- | --- |
| **TITLE** | | |  |
| Title | 1 | Identify the report as a systematic review. | 1 |
| **ABSTRACT** | | |  |
| Abstract | 2 | See the PRISMA 2020 for Abstracts checklist. (made as per the Journal guidelines) | 2 |
| **INTRODUCTION** | | |  |
| Rationale | 3 | Describe the rationale for the review in the context of existing knowledge. | 3 |
| Objectives | 4 | Provide an explicit statement of the objective(s) or question(s) the review addresses. | 3 |
| **METHODS** | | |  |
| Eligibility criteria | 5 | Specify the inclusion and exclusion criteria for the review and how studies were grouped for the syntheses. | 4 |
| Information sources | 6 | Specify all databases, registers, websites, organisations, reference lists and other sources searched or consulted to identify studies. Specify the date when each source was last searched or consulted. | 4 |
| Search strategy | 7 | Present the full search strategies for all databases, registers and websites, including any filters and limits used. | Table S3 |
| Selection process | 8 | Specify the methods used to decide whether a study met the inclusion criteria of the review, including how many reviewers screened each record and each report retrieved, whether they worked independently, and if applicable, details of automation tools used in the process. | 4 |
| Data collection process | 9 | Specify the methods used to collect data from reports, including how many reviewers collected data from each report, whether they worked independently, any processes for obtaining or confirming data from study investigators, and if applicable, details of automation tools used in the process. | 4, 5 |
| Data items | 10a | List and define all outcomes for which data were sought. Specify whether all results that were compatible with each outcome domain in each study were sought (e.g., for all measures, time points, analyses), and if not, the methods used to decide which results to collect. | 4, Table 1 |
|  | 10b | List and define all other variables for which data were sought (e.g., participant and intervention characteristics, funding sources). Describe any assumptions made about any missing or unclear information. | 4 |
| Study risk of bias assessment | 11 | Specify the methods used to assess risk of bias in the included studies, including details of the tool(s) used, how many reviewers assessed each study and whether they worked independently, and if applicable, details of automation tools used in the process. | 5, Table S4 |
| Effect measures | 12 | Specify for each outcome the effect measure(s) (e.g. risk ratio, mean difference) used in the synthesis or presentation of results. | 5 |
| Synthesis methods | 13a | Describe the processes used to decide which studies were eligible for each synthesis (e.g. tabulating the study intervention characteristics and comparing against the planned groups for each synthesis (item #5)). | 5, Table 1 |
|  | 13b | Describe any methods required to prepare the data for presentation or synthesis, such as handling of missing summary statistics, or data conversions. | NA |
|  | 13c | Describe any methods used to tabulate or visually display results of individual studies and syntheses. | 4 |
|  | 13d | Describe any methods used to synthesize results and provide a rationale for the choice(s). If meta-analysis was performed, describe the model(s), method(s) to identify the presence and extent of statistical heterogeneity, and software package(s) used. | 5 |
|  | 13e | Describe any methods used to explore possible causes of heterogeneity among study results (e.g. subgroup analysis, meta-regression). | 6 |
|  | 13f | Describe any sensitivity analyses conducted to assess robustness of the synthesized results. | 6 |
| Reporting bias assessment | 14 | Describe any methods used to assess risk of bias due to missing results in a synthesis (arising from reporting biases). | NA |
| Certainty assessment | 15 | Describe any methods used to assess certainty (or confidence) in the body of evidence for an outcome. | NA |
| **RESULTS** | | |  |
| Study selection | 16a | Describe the results of the search and selection process, from the number of records identified in the search to the number of studies included in the review, ideally using a flow diagram. | Figure-1 |
|  | 16b | Cite studies that might appear to meet the inclusion criteria, but which were excluded, and explain why they were excluded. | 6, Table 1 |
| Study characteristics | 17 | Cite each included study and present its characteristics. | Table-1 |
| Risk of bias in studies | 18 | Present assessments of risk of bias for each included study. | Table S4 |
| Results of individual studies | 19 | For all outcomes, present, for each study: (a) summary statistics for each group (where appropriate) and (b) an effect estimate and its precision (e.g. confidence/credible interval), ideally using structured tables or plots. | Table 1, Figure 2,3 |
| Results of syntheses | 20a | For each synthesis, briefly summarise the characteristics and risk of bias among contributing studies. | 5 |
|  | 20b | Present results of all statistical syntheses conducted. If meta-analysis was done, present for each the summary estimate and its precision (e.g. confidence/credible interval) and measures of statistical heterogeneity. If comparing groups, describe the direction of the effect. | 5, 6 Figure 2, 3 |
|  | 20c | Present results of all investigations of possible causes of heterogeneity among study results. | 6 |
|  | 20d | Present results of all sensitivity analyses conducted to assess the robustness of the synthesized results. | NA |
| Reporting biases | 21 | Present assessments of risk of bias due to missing results (arising from reporting biases) for each synthesis assessed. | NA |
| Certainty of evidence | 22 | Present assessments of certainty (or confidence) in the body of evidence for each outcome assessed. | NA |
| **DISCUSSION** | | |  |
| Discussion | 23a | Provide a general interpretation of the results in the context of other evidence. | 6 |
|  | 23b | Discuss any limitations of the evidence included in the review. | 7 |
|  | 23c | Discuss any limitations of the review processes used. | 8 |
|  | 23d | Discuss implications of the results for practice, policy, and future research. | 9 |
| **OTHER INFORMATION** | | |  |
| Registration and protocol | 24a | Provide registration information for the review, including register name and registration number, or state that the review was not registered. | 4 |
|  | 24b | Indicate where the review protocol can be accessed, or state that a protocol was not prepared. | 4 |
|  | 24c | Describe and explain any amendments to information provided at registration or in the protocol. | NA |
| Support | 25 | Describe sources of financial or non-financial support for the review, and the role of the funders or sponsors in the review. | 9 |
| Competing interests | 26 | Declare any competing interests of review authors. | 9 |
| Availability of data, code and other materials | 27 | Report which of the following are publicly available and where they can be found: template data collection forms; data extracted from included studies; data used for all analyses; analytic code; any other materials used in the review. | 9 |

**Table S2. The adjusted search terms as per searched electronic databases [as of 15.05.2024]**

| **Database** | **No** | **Search Query** | **Results** | |
| --- | --- | --- | --- | --- |
| **PubMed** | | | |  |
|  | #1 | ("Salmonella typhi"[All Fields] OR "typhoid"[All Fields]) AND ("antibiotic resistance"[All Fields] OR "antimicrobial resistance"[All Fields] OR "drug resistance"[All Fields]) AND "Pakistan"[All Fields] AND ("2014"[All Fields] OR "2015"[All Fields] OR "2016"[All Fields] OR "2017"[All Fields] OR "2018"[All Fields] OR "2019"[All Fields] OR "2020"[All Fields] OR "2021"[All Fields] OR "2022"[All Fields] OR "2023"[All Fields] OR "2024"[All Fields]) | 370 | |
| **Science Direct** | | | |  |
|  | #1 | ("Salmonella" OR "Typhoid fever") AND ("Antibiotic resistance" OR "Antimicrobial resistance" OR "Drug resistance") AND "Pakistan" AND (2014-2024) NOT "salmonella" | 282 | |
| **Embase** | | | |  |
|  | #1 | ('salmonella':ti,ab,kw OR 'typhoid fever':ti,ab,kw) AND ('antibiotic resistance':ti,ab,kw OR 'antimicrobial resistance':ti,ab,kw OR 'drug resistance':ti,ab,kw) AND 'pakistan':ti,ab,kw AND [2014-2024]/py NOT 'salmonalla' | 523 | |
| **Web of Science** | | | |  |
|  | #1 | TS=("Salmonella" OR "Typhoid fever") AND TS=("Antibiotic resistance" OR "Antimicrobial resistance" OR "Drug resistance") AND TS=("Pakistan") AND PY=(2014-2024) NOT TS=("salmone") | 298 | |
| **Google Scholar** | | | |  |
|  | #1 | ("Salmonella" OR "Typhoid fever") AND ("Antibiotic resistance" OR "Antimicrobial resistance" OR "Drug resistance") AND "Pakistan" AND (2014..2024) | 380 | |
| **PakMediNet** | | | | |
|  |  | Salmonella OR Typhoid fever AND Antibiotic resistance OR Antimicrobial resistance OR Drug resistance AND Pakistan 2014-2024 | 10 | |

**Table S3. Inclusion and Exclusion criteria**

**Research Question: What is the prevalence and trend of antibiotic resistance in Salmonella typhi in Pakistan from 2014 to 2024, and which antibiotics show the highest and lowest resistance rates?**

| **Inclusion** | | **Exclusion** |
| --- | --- | --- |
| **Participants** | Patients diagnosed with Salmonella infection in Pakistan | None |
| **Intervention** | None |  |
| **Outcome** | Prevalence of resistance to different antibiotics, MDR and XDR | None |
| **Study Designs** | Observational studies (cross-sectional, cohort, case-control) and surveillance reports | Without clear resistance data, studies were not conducted in Pakistan and were non-peer-reviewed. |
|  | Date of Search- 15^th^ May 2024  Published articles and preprint data in the English language | Unavailable full-text articles not in English language |

**Table S4. Quality assessment using a JBI quality assessment tool for prevalence studies.**

| **Study** | **D1** | **D2** | **D3** | **D4** | **D5** | **D6** | **D7** | **D8** | **D9** | **Overall Quality of the Study** |
| --- | --- | --- | --- | --- | --- | --- | --- | --- | --- | --- |
| Yousaf 2024[13] | Yes | Yes | No | Yes | Unclear | Yes | Unclear | Yes | Unclear | Moderate |
| Umair 2020[16] | Yes | Yes | No | Yes | Yes | NA | Yes | Yes | Yes | High |
| Aslam 2021[17] | Yes | Yes | Yes | Yes | Yes | Yes | Unclear | Yes | Unclear | Moderate |
| Saeed 2019[15] | Yes | No | No | Yes | Yes | Yes | Yes | Yes | Unclear | Moderate |
| Saeed 2020[14] | Yes | Yes | No | Yes | Yes | Yes | Yes | Unclear | Unclear | Moderate |
| Hussain 2022[18] | Yes | Yes | Yes | Yes | Yes | Yes | Yes | Yes | Unclear | High |
| Aslam 2024[19] | Yes | Yes | Yes | Yes | Unclear | Yes | Yes | Yes | Unclear | Moderate |
| Khan 2024[20] | Yes | Yes | Yes | Yes | Yes | Yes | Yes | Yes | Unclear | High |
| Laghari, 2019[21] | Yes | Yes | Yes | Yes | Unclear | Yes | Yes | Yes | Unclear | Moderate |
| Ullah 2024[22] | Yes | No | Yes | Yes | Yes | Unclear | Yes | Yes | Yes | Moderate |
| Ejaz 2022[23] | No | Yes | No | Unclear | No | Yes | Yes | No | Yes | Low |
| Anjum 2021[24] | Unclear | Yes | Unclear | Yes | Unclear | Yes | Yes | Yes | No | Low |
| Zahid2022[36] | Yes | No | Yes | Yes | No | No | No | Yes | No | Low |
| Batool 2021[26] | Yes | Unclear | No | Yes | No | Yes | No | No | Yes | Low |
| Ahmad,2022[27] | Yes | No | Yes | Yes | No | No | No | Yes | No | Low |
| Sattar, 2020[28] | Yes | No | Yes | Yes | Yes | Unclear | Yes | Yes | Yes | Moderate |
| Hussain 2019[29] | Yes | Unclear | No | Yes | No | Yes | No | No | Yes | Low |
| Izhar 2020[30] | Yes | Unclear | No | Yes | No | Yes | No | No | Yes | Low |
| Ahmad,2020[31] | Yes | Yes | Yes | Yes | Unclear | Yes | Yes | Yes | Unclear | Moderate |
| Sherazi 2023[32] | Yes | No | Yes | Yes | Yes | Unclear | Yes | Yes | Yes | Moderate |
| Khan,2023[33] | Yes | Unclear | Yes | Yes | Yes | Unclear | Yes | Yes | Yes | High |
| Baig,2023[34] | Yes | Unclear | No | Yes | No | Yes | No | No | Yes | Low |
| Shah2024[35] | Yes | Unclear | No | Yes | No | Yes | No | No | Yes | High |
| Ejaz 2022[23] | Yes | Unclear | Yes | Yes | Yes | Unclear | Yes | Yes | Yes | Moderate |
| Zahid, 2022[36] | Yes | Yes | Yes | Yes | Yes | Yes | Yes | Yes | Yes | High |

**References:**

1. Saeed, M., et al., *Extended-spectrum beta-lactamases producing extensively drug-resistant Salmonella Typhi in Punjab, Pakistan.* J Infect Dev Ctries, 2020. **14**(2): p. 169-176.

2. Aslam, A., et al., *Trends of Antimicrobial Resistance in Typhoidal Strains of Salmonella in a Tertiary Care Hospital in Pakistan.* Cureus, 2021. **13**(1): p. e12664.

3. Aslam, S., et al., *Clinical presentation and outcome of enteric fever in adult patients with cancer: a perspective from Pakistan.* Access Microbiol, 2024. **6**(5).

4. Ejaz, A., et al., *Frequency and antimicrobial resistance patterns of Salmonella enterica isolates in a tertiary care setting.* Pakistan Journal of Medical & Health Sciences, 2022. **16**(05): p. 11-11.

5. Zahid, S., et al., *Strains of Typhoid Salmonella in Pakistan Causing an Impending Threat for Drug-Resistant.* Pakistan Journal of Medical & Health Sciences, 2022. **16**(03): p. 336-336.

6. Batool, A., et al., *Prevalence of Multi-Drug Resistant and Extensively Drug-Resistant Salmonella enterica Serovar Typhi Recovered from Pediatrics’ Septicemia Patients in Lahore.* Pakistan Journal of Medical and Health Sciences, 2021. **15**(4): p. 843-845.

7. Baig, U., S.M. Mehdi, and N. Iftikhar, *A pattern of antibiotic drug resistance of Salmonella Typhi and Salmonella Paratyphi among children with enteric fever in a tertiary care hospital in Lahore, Pakistan.* Croatian Medical Journal, 2023. **64**(4): p. 256.

8. Latif, S., et al., *Extensively drug resistant typhoid fever seen at tertiary care hospital in Lahore.* Infectious Diseases Journal of Pakistan, 2019. **28**(3): p. 51-54.

9. Rasheed, F., et al., *Trend of antimicrobial resistance among Salmonella typhi isolated from the pediatric population presenting at a tertiary care hospital in Lahore.* Biomedica, 2023. **39**(2): p. 61-66.

10. Shabbir, T., et al., *Frequency of the multi and extensively drug-resistant Salmonella Typhi in a health care facility.* Journal of Fatima Jinnah Medical University, 2022. **16**(3): p. 138-142.

11. Zahid, I., et al., *Antibiotyping and genotyping of extensively drug‐resistant (XDR) Salmonella sp. isolated from clinical samples of Lahore, Pakistan.* Journal of applied microbiology, 2022. **132**(1): p. 633-641.

12. Ahmad, M., N. Shah, and M.A. Siddiqui, *Frequency and Antibiotics sensitivity pattern of culture-positive Salmonella Typhi in Children.* J Coll Physicians Surg Pak, 2023. **33**(03): p. 303-7.

13. Zakir, M., et al., *Emerging trends of multidrug-resistant (MDR) and extensively drug-resistant (XDR) Salmonella Typhi in a tertiary care Hospital of Lahore, Pakistan.* Microorganisms, 2021. **9**(12): p. 2484.

14. Yousaf, M., et al., *Antibiotic Resistance in Salmonella typhi Strains Isolated From Patients in Pakistan: A Hospital Database Study.* Cureus, 2024. **16**(4): p. e58240.

15. Khan, M., et al., *A comparable risk of extensively drug-resistant typhoid fever in the pediatric cohort during the COVID-19 pandemic.* Int J Health Sci (Qassim), 2024. **18**(1): p. 24-28.

16. Ullah, R., et al., *Comprehensive Analysis of Salmonella Species Antibiogram and Evolving Patterns in Empirical Therapy: Insights From Tertiary Care Hospitals in Peshawar, Pakistan.* Cureus, 2024. **16**(3): p. e57110.

17. Ahmad, S., et al., *Trends in antibiotic susceptibility of enteric fever isolates among children attending a tertiary care hospital of Peshawar, KP.* Journal of Rehman Medical Institute, 2020. **6**(3): p. 20-23.

18. Sherazi, F., et al., *MULTIPLE DRUG RESISTANCE, EXTENSIVELY DRUG RESISTANCE TYPHOID FEVER AND DISEASE SPECTRUM IN PEDIATRIC POPULATION PRESENTING WITH FEVER WITHOUT LOCALIZING SIGNS (FWLS): A CROSS SECTIONAL STUDY.* KJMS, 2023. **16**(2): p. 103.

19. Khan, A.M., et al., *Typhoid-Related Positive Pattern Of Antibiotic Sensitivity In Blood Cultures A Cross-Sectional Study.* Journal of Bacha Khan Medical College, 2023. **4**(02): p. No. 14-17.

20. Shah, M.W., I. Ali, and R. Zaman, *Antimicrobial Susceptibility Patterns of Salmonella Typhi and Salmonella Paratyphi in Tertiary Care Hospitals.* Pakistan Journal of Medical & Health Sciences, 2024. **18**(01): p. 59-59.

21. Umair, M. and S.A. Siddiqui, *Antibiotic Susceptibility Patterns of Salmonella Typhi and Salmonella Paratyphi in a Tertiary Care Hospital in Islamabad.* Cureus, 2020. **12**(9): p. e10228.

22. Saeed, N., M. Usman, and E.A. Khan, *An Overview of Extensively Drug-resistant Salmonella Typhi from a Tertiary Care Hospital in Pakistan.* Cureus, 2019. **11**(9): p. e5663.

23. Anjum, M., et al., *Clinical Spectrum, Laboratory Profile and Antibiotic Susceptibility Pattern of Children with Enteric Fever at a Tertiary Care Hospital of Karachi.* Journal of Islamic International Medical College (JIIMC), 2021. **16**(1): p. 4-9.

24. Javed, H., et al., *Metallo-beta-lactamase producing Escherichia coli and Klebsiella pneumoniae: a rising threat for hospitalized children.* JPMA, 2016. **66**(1068).

25. Usman Qamar, M., et al., *The present danger of New Delhi metallo-β-lactamase: A threat to public health.* Future microbiology, 2020. **15**(18): p. 1759-1778.

26. *<Prevalence of Multi-Drug Resistant and Extensively Drug-Resistant Salmonella enterica......pdf>.*

27. Izhar, K., et al., *Extensively drug-resistant Salmonella Typhi XDR infection at Rawalpindi medical university and allied hospitals.* Journal of Rawalpindi Medical College, 2020. **24**(4).

28. Azmatullah, A., et al., *Systematic review of the global epidemiology, clinical and laboratory profile of enteric fever.* Journal of global health, 2015. **5**(2).

29. Hussain, A., et al., *Typhoidal Salmonella strains in Pakistan: an impending threat of extensively drug-resistant Salmonella Typhi.* European Journal of Clinical Microbiology & Infectious Diseases, 2019. **38**: p. 2145-2149.

30. Shaikh, A.A., A. Shaikh, and A. Tahir, *Antimicrobial resistance trends of typhoidal salmonellae in southern Pakistan.* Rawal Medical Journal, 2019. **44**(1): p. 7-7.

31. Laghari, G.S., et al., *Antimicrobial Susceptibility Patterns of Salmonella Species in Southern Pakistan.* Cureus, 2019. **11**(4): p. e4379.

32. AHMAD, F., et al., *Antibiotic Sensitivity Pattern in Blood Culture Positive Typhoid Fever.* Methods, 2020.

33. Das, J.K., et al., *Trends, associations, and antimicrobial resistance of Salmonella typhi and paratyphi in Pakistan.* The American journal of tropical medicine and hygiene, 2018. **99**(3 Suppl): p. 48.

34. Batool, R., et al., *Efficacy of typhoid vaccines against culture-confirmed Salmonella Typhi in typhoid endemic countries: a systematic review and meta-analysis.* Lancet Glob Health, 2024. **12**(4): p. e589-e598.

35. Gibani, M.M., C. Britto, and A.J. Pollard, *Typhoid and paratyphoid fever: a call to action.* Current opinion in infectious diseases, 2018. **31**(5): p. 440-448.

36. Zahid, S., et al., *Strains of Typhoid Salmonella in Pakistan Causing an Impending Threat for Drug-Resistant.* Pakistan Journal of Medical and Health Sciences, 2022. **16**(3): p. 336-337.

37. Hasan, R., et al., *Antibiotic resistance among Salmonella enterica serovars Typhi and Paratyphi A in Pakistan (2001-2006).* The Journal of Infection in Developing Countries, 2008. **2**(04): p. 289-294.

38. Sajib, M.S., et al., *Tracking the emergence of azithromycin resistance in multiple genotypes of typhoidal salmonella.* MBio, 2021. **12**(1): p. 10.1128/mbio. 03481-20.

39. Akram, J., et al., *Extensively drug‐resistant (XDR) typhoid: evolution, prevention, and its management.* BioMed Research International, 2020. **2020**(1): p. 6432580.

40. Rasheed, M., S.S. Hasan, and S.I. Ahmed, *Extensively drug-resistant typhoid fever in Pakistan.* The Lancet Infectious Diseases, 2019. **19**(3): p. 242-243.

41. Ashraf Hussain, M., et al., *Extensively Drug-Resistant Typhoidal Salmonellae: Are These Bugs Swarming Into Suburban and Rural Areas of Pakistan?* Cureus, 2022. **14**(6): p. e26189.

42. Mazhar, N., et al., *Causative Organisms in Neonatal Sepsis and Their Antibiotic Sensitivity and Resistance Pattern.* Pakistan Journal of Medical & Health Sciences, 2022. **16**(03): p. 276-276.

43. Sattar, A., et al., *Current trends in antimicrobial susceptibility pattern of Salmonella typhi and paratyphi.* Rawal Medical Journal, 2020. **45**(2): p. 291-4.

44. Medalla, F., et al., *Increased Incidence of Antimicrobial-Resistant Nontyphoidal Salmonella Infections, United States, 2004-2016.* Emerg Infect Dis, 2021. **27**(6): p. 1662-1672.

45. Manafi, L., J. Aliakbarlu, and H. Dastmalchi Saei, *Antibiotic resistance and biofilm formation ability of Salmonella serotypes isolated from beef, mutton, and meat contact surfaces at retail.* Journal of Food Science, 2020. **85**(8): p. 2516-2522.

46. Narimisa, N., S. Razavi, and F. Masjedian Jazi, *Prevalence of antibiotic resistance in Salmonella Typhimurium isolates originating from Iran: a systematic review and meta-analysis.* Front Vet Sci, 2024. **11**: p. 1388790.

47. Carey, M.E., et al., *Spontaneous Emergence of Azithromycin Resistance in Independent Lineages of Salmonella Typhi in Northern India.* Clinical Infectious Diseases, 2021. **72**(5): p. e120-e127.

48. Qureshi, S., et al., *Response of extensively drug resistant Salmonella Typhi to treatment with meropenem and azithromycin, in Pakistan.* PLoS Negl Trop Dis, 2020. **14**(10): p. e0008682.

49. Walker, J., et al., *Assessing the global risk of typhoid outbreaks caused by extensively drug resistant Salmonella Typhi.* Nature Communications, 2023. **14**(1): p. 6502.
